# *T. gondii* infection induces IL-1R dependent chronic cachexia and perivascular fibrosis in the liver and skeletal muscle

**DOI:** 10.1038/s41598-020-72767-0

**Published:** 2020-09-24

**Authors:** Stephanie J. Melchor, Jessica A. Hatter, Érika A. LaTorre Castillo, Claire M. Saunders, Kari A. Byrnes, Imani Sanders, Daniel Abebayehu, Thomas H. Barker, Sarah E. Ewald

**Affiliations:** 1 Department of Microbiology, Immunology, and Cancer Biology and The Carter Immunology Center, University of Virginia School of Medicine, Charlottesville, VA USA; 2 Department of Pharmacology, University of Virginia School of Medicine, Charlottesville, VA USA; 3 Ponce Health Sciences University, Ponce, PR USA; 4 Department of Biomedical Engineering, University of Virginia School of Medicine, Charlottesville, VA USA

**Keywords:** Innate immunity, Interleukins, Immunology, Chronic inflammation

## Abstract

Cachexia is a progressive muscle wasting disease that contributes to death in a wide range of chronic diseases. Currently, the cachexia field lacks animal models that recapitulate the long-term kinetics of clinical disease, which would provide insight into the pathophysiology of chronic cachexia and a tool to test therapeutics for disease reversal. *Toxoplasma gondii *(*T. gondii*) is a protozoan parasite that uses conserved mechanisms to infect rodents and human hosts. Infection is lifelong and has been associated with chronic weight loss and muscle atrophy in mice. We have recently shown that *T. gondii*-induced muscle atrophy meets the clinical definition of cachexia. Here, the longevity of the *T. gondii*-induced chronic cachexia model revealed that cachectic mice develop perivascular fibrosis in major metabolic organs, including the adipose tissue, skeletal muscle, and liver by 9 weeks post-infection. Development of cachexia, as well as liver and skeletal muscle fibrosis, is dependent on intact signaling through the type I IL-1R receptor. IL-1α is sufficient to activate cultured fibroblasts and primary hepatic stellate cells (myofibroblast precursors in the liver) in vitro, and IL-1α is elevated in the sera and liver of cachectic, suggesting a mechanism by which chronic IL-1R signaling could be leading to cachexia-associated fibrosis.

## Introduction

Cachexia is defined as the loss of 5% of lean body mass in less than 6 months, accompanied by at least three of the following symptoms: weakness, fatigue, adipose tissue loss, abnormal blood biochemistry, and/or anorexia^1^. Cachexia occurs in conjunction with a primary chronic disease, and prevalence can range from 5–15% in chronic heart failure and chronic pulmonary disease to over 80% in advanced cancer patients^2,3^. Beyond impairing quality of life, cachexia limits the effectiveness and duration of therapeutic interventions and can directly cause death in chronically ill patients^4–6^. Currently, there are no broadly efficacious therapeutics for cachexia. Dietary supplementation and anabolic steroids have largely failed to reverse clinical cachexia^7^. Circulating IL-1, IL-6, TNF-α, and IFN-γ are a conserved inflammatory signature across etiologies of cachexia; however, TNF-α blockade has failed to halt or reverse cachexia in the clinic^8^. More recently a first-in-class monoclonal antibody against IL-1α has been shown to increase lean body mass and quality of life in patients with metastatic cancers^9^. While these data suggest that there may be therapeutic value in targeting the IL-1 pathway for cachexia, a disease-matched study of cachectic and weight stable patients has not yet been performed.

Current animal models of cachexia recapitulate acute cachectic weight loss, but fail to model many aspects of long-term cachexia progression. Surgical interventions including cardiac, gastric, and renal obstructive surgeries are lethal after a rapid period of weight loss. Tumor models take longer to develop but have a similar 1–2 week window of weight loss before tumor growth is lethal. Interventions like low level endotoxin injection are transient, and in a new cachexia model using lymphocytic choriomeningitis virus (LCMV) infection, mice recover weight once the virus is cleared^10,11^. Although these studies have uncovered important aspects of acute cachexia biology, longer-term models may reveal novel pathways of homeostatic dysfunction and potential therapeutic targets. Cachexia or cachexia-like disease is extraordinarily common in parasitic infection; however, animal models of parasite-induced cachexia have received comparatively little attention in the modern molecular era^12^. We have recently shown that both oral^13^ and intraperitoneal^14^ infection with the protozoan parasite *Toxoplasma gondii* (*T. gondii*) leads to a sustained loss of muscle mass that meets the current clinical definition of cachexia, including long-term muscle and fat wasting, fatigue, and elevated circulating cytokines^1^*. T. gondii* is a protozoan parasite that naturally infects humans and mice. During acute infection (the first 3 weeks of infection) the parasite can be found in most tissues in the body. Immune competent hosts largely clear systemic infection, but low levels of *T. gondii* tissue cysts persist for the life of the host in the brain and skeletal muscle, among other tissues^15^. Controlling chronic infection depends on a sustained, low grade inflammatory response and TH1 immunity, characterized by IL-12, IFN-γ, and CD4^+^ and CD8^+^ T cells.

We hypothesized that the robust and reproducible nature of *T. gondii* infection-induced cachexia would yield novel insight into the pathophysiology of chronic cachexia. The intraperitoneal infection model was chosen to limit the influence of commensal microbiota on cachexia progression, as previous reports have shown that commensal bacteria breach the intestinal barrier in acute oral *Toxoplasma* infection and exhibit a sustained shift towards Gram-negative populations^13,16–18^. Unlike what has been observed in animal models of acute anorexia cachexia, lipolysis and fat browning pathways were not activated. Instead, the longevity of our model revealed that cachectic mice develop perivascular fibrosis in major metabolic tissues, including the visceral white adipose tissue, skeletal muscle, and the liver. Chronic cachexia and cachexia-associated liver and skeletal muscle fibrosis was IL-1R dependent. This increase in fibrosis was not due to parasite overgrowth, as infection levels were similar between IL-1R^−/−^ and wildtype mice. IL-1α levels were elevated in sera and liver lysates of cachectic mice; in vitro, IL-1α or IL-1β were sufficient to promote fibroblast contractility and expression of smooth muscle actin. These findings are consistent with a novel role for the IL-1R signaling axis as a driver of chronic cachexia and the development of cachexia-associated fibrosis during *T. gondii* infection.

## Results

### *T. gondii* infection leads to sustained cachexia in mice

**Figure 1:**
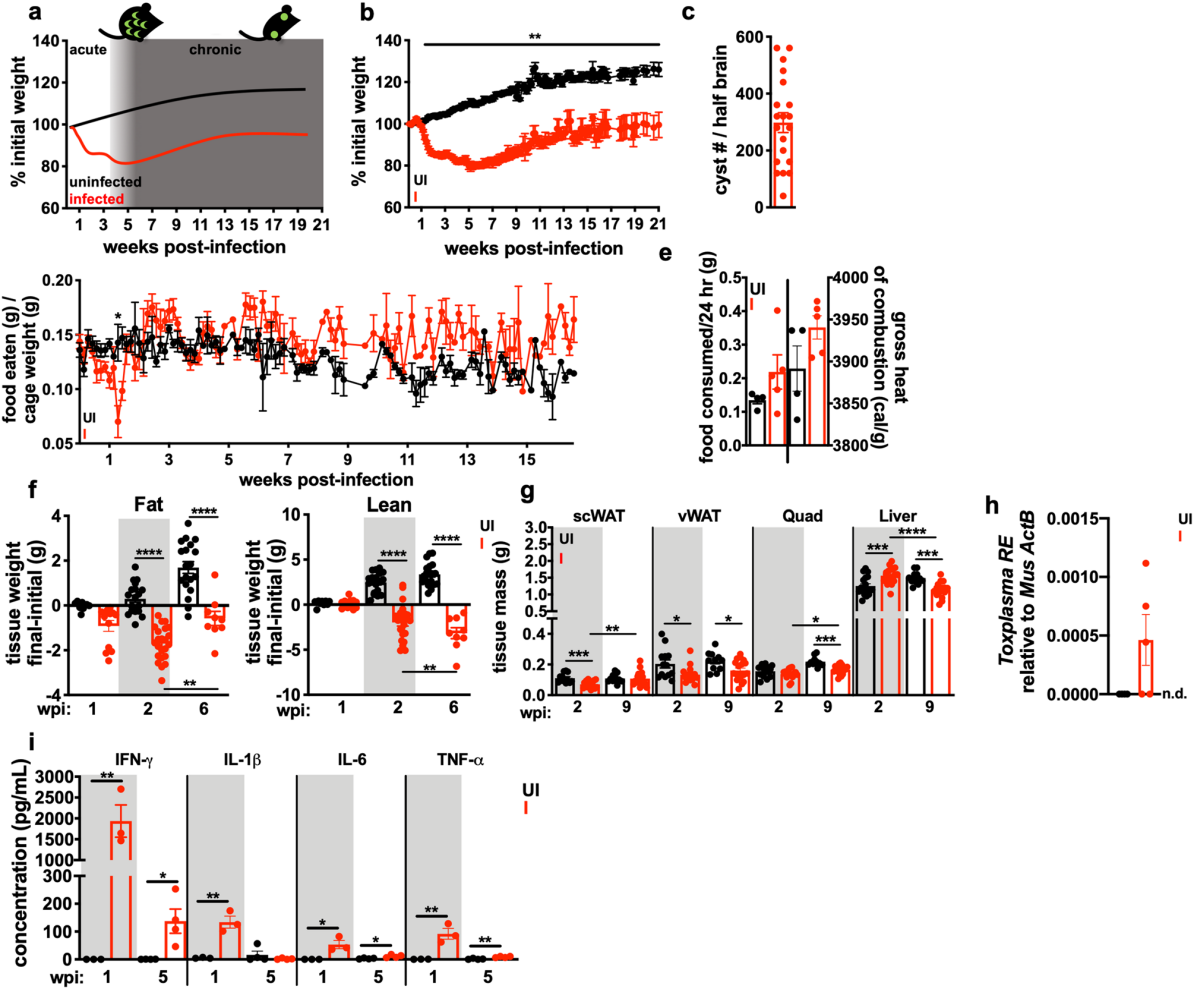
Chronic *Toxoplasma* infection causes sustained cachexia in mice. 10–14 week old C57BL/6J mice were intraperitoneally infected with 10 Me49-GFP-luciferase *T. gondii* cysts (I, red) or mock injected with PBS (UI, black). (**a**) Schematic of weight loss relative to parasite distribution. The acute phase of infection (white) is dominated by *Toxoplasma tachyzoites* (green crescents) which spread systemically, infecting most tissues in the body. 4–6 weeks post-infection (wpi), systemic infection is largely cleared and parasites are driven to the chronic tissue cyst form (green circles). (**b**) Mice lose up to 20% of their initial body mass in the first 4 wpi and fail to regain weight relative to uninfected controls. N = 35–45 mice pooled from 3 independent experiments. (**c**) *Dolichos biflorous* positive *T. gondii* cysts per half brain at 5–9 wpi. N = 19 pooled from 6 independent experiments. (**d**) Daily food intake per cage normalized to pooled weight of the mice in the cage measured every 24 h. N = 7–9 cages per group, pooled from 3 independent experiments. (**e**) Mice were individually housed for 24 h at 10 wpi and food intake over 24 h was determined by weight (left) and caloric content of fecal pellets were determined by bomb calorimetry (right). N = 4 mice per group. (**f**) Echo MRI quantification of fat (left) and lean (right) tissue mass at 2 or 6 wpi. N = 28–45 mice per group, representative of 3 independent experiments. (**g**) Inguinal subcutaneous white adipose tissue (scWAT), epididymal visceral white adipose tissue (vWAT), quadriceps (Quad) and liver weights at 2 wpi or 9 wpi. N = 12–18 mice per group, pooled from 3 experiments. (**h**) Quantity of *T. gondii* DNA relative to host beta-actin in the tibialis anterior muscle at 9 wpi. N = 4–5 mice per group. *n.d. *not detectable. (**i**) Serum cytokines measured by Luminex at 1 or 5 wpi. N = 3–4 mice per group, representative of 2 experiments. Error bars are standard error of the mean. *P < 0.05; **P < 0.01; ***P < 0.001, ****P < 0.0001 by unpaired Student’s *t* test.

Oral infection with *T. gondii* causes severe intestinal inflammation, leaky gut, and commensal-induced inflammation during the first 2 weeks of infection^16,19,20^. We previously demonstrated that mice orally infected with *T. gondii* develop chronic cachexia that was sustained even after intestinal inflammation resolved^13^. We have also shown that *T. gondii-*induced cachexia occurs when the gastrointestinal tract is bypassed entirely using intraperitoneal infection^14^. To understand the molecular mechanisms controlling chronic cachexia, 10–14 week old male C57BL/6J mice were intraperitoneally injected with 10 Type II *T. gondii* (Me49 strain) bradyzoite tissue cysts. *T. gondii*-infected mice lost 15–20% of their initial body mass during the first 3 weeks of infection (Fig. 1a,b), and remained significantly wasted compared to uninfected controls for up to 21 weeks (Fig. 1b). Chronic infection was confirmed by counting cysts in brain homogenate at 9 weeks post-infection (Fig. 1c). Infected female mice exhibited similar kinetics of weight loss during infection with *T. gondii* (Supp. Figure 1a). Although infected mice went through a period of acute anorexia 8–10 days post-infection, they regained eating relative to uninfected controls, indicating that sustained weight loss was not due to prolonged anorexia (Fig. 1d). Growth stunting or developmental malnutrition is a distinct disease from cachexia, so to avoid inducing this biology, 10–14 week old, mature adult mice were used in all experiments. Infected mice trended towards eating more chow per 24-h period than uninfected mice, although this was not significant (Fig. 1d,e, left). Bomb calorimetry on fecal pellets confirmed that infected mice were absorbing a similar number of calories as uninfected mice (Fig. 1e, right).

Loss of lean muscle mass is the primary diagnostic marker of cachexia. By 2 weeks post-infection, infected mice had a significant reduction in both fat (Fig. 1f, left) and lean (Fig. 1f, right) body mass by EchoMRI Whole Body Composition Analysis. Importantly, while fat mass partially recovered by 6 weeks post-infection, lean body mass wasting progressed from 2 to 6 weeks post-infection (Fig. 1f). When individual tissues were dissected and weighed, we found that inguinal subcutaneous white adipose tissue (scWAT) and epigonadal visceral white adipose tissue (vWAT) were significantly reduced at 2 weeks post-infection. Acute hepatomegaly was also observed, consistent with published reports of acute *T. gondii* infection in the liver (Fig. 1g)^21^. By 9 weeks post-infection, scWAT had recovered in mass but vWAT, quadriceps muscle (quad), and liver were significantly reduced in size relative to uninfected littermate controls (Fig. 1g). Gastrocnemius (GA), tibialis anterior (TA), and extensor digitorus longum (EDL) muscles were also weighed. The TA and EDL were significantly smaller in infected mice relative to uninfected; the GA from infected mice trended smaller, although this was not significant due to variability in GA mass (Supp. Figure 1b). *T. gondii* burden was measured in the tibialis anterior at 10 weeks post-infection by qPCR of parasite DNA (Fig. 1h). Consistent with previous reports that parasite burden is low in skeletal muscle, *Toxoplasma* genomic DNA was detected in 3 out of 5 infected samples, suggesting that while there may be a chronic parasite presence in muscle tissue, this is not sufficient to explain such widespread muscle wasting^22^. Elevated circulating inflammatory cytokines are a hallmark of cachexia that contribute to disease pathology^23^. At 1 week post-infection, when *T. gondii* infection is systemic, mice had elevated circulating IFN-γ, IL-1β, IL-6, and TNF-α compared to uninfected controls (Fig. 1i, grey background). Although TNF-α, IL-6, and IFN-γ were reduced by 5 weeks post-infection, they were still significantly elevated relative to uninfected controls (Fig. 1i, white background). Based on these data, intraperitoneal *T. gondii* infection causes loss of adipose tissue, progressive muscle loss, transient anorexia, and elevated innate circulating cytokines, consistent with the current clinical definition of cachexia^1^.

### *T. gondii*-induced chronic cachexia is independent of non-shivering thermogenesis, insulin resistance, and elevated lipolysis

**Figure 2:**
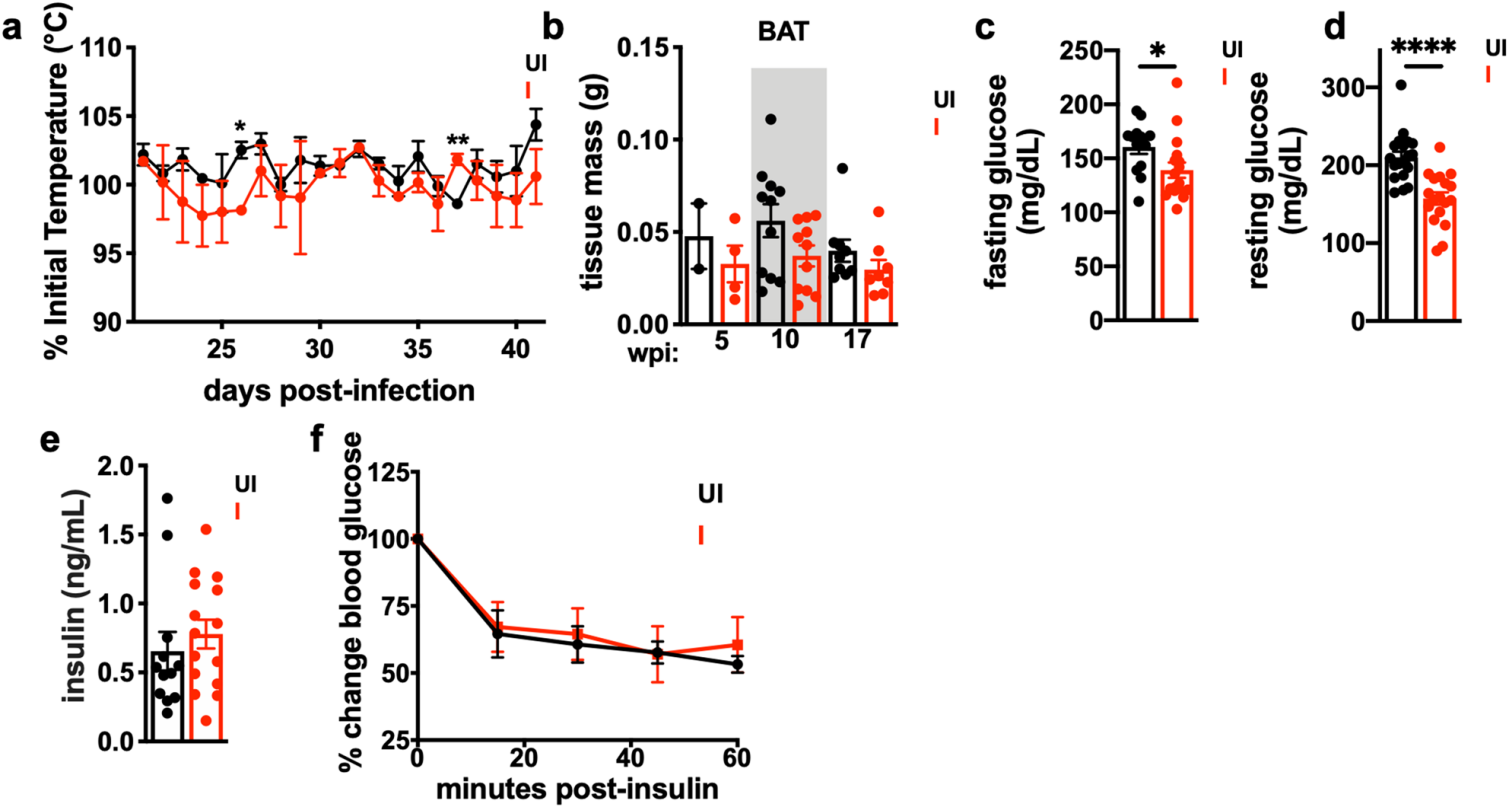
*T. gondii*-induced chronic cachexia occurs independently of non-shivering thermogenesis and insulin resistance. (**a**) Male and female mice were subcutaneously injected with telemetric temperature probes at 11 days post-infection, and temperature was monitored daily. (**b**) Supraclavicular brown adipose tissue (BAT) weights at 5, 10, or 17 wpi. N = 2–11 mice per group. (**c**,**d**) blood glucose was collected by tail snip and either measured after 4 h of fasting at 9 wpi (**c**, N = 13–17, pooled between 3 independent experiments) or after random feeding at 7–14 wpi (**d**, N = 18–19 mice per group, pooled between 2 independent experiments) by glucometer. (**e**) Serum insulin levels measured by ELISA at 7–10 wpi. N = 12–15 mice per group, pooled between two independent experiments. (**f**) Random-fed mice were intraperitoneally injected with 0.75 U insulin/kg body weight at 7–14 wpi, and blood glucose was measured every 15 min. N = 7–9 mice per group, pooled between two independent experiments. *P < 0.05; **P < 0.01; ***P < 0.001, ****P < 0.0001 by unpaired Student’s *t* test.

Non-shivering thermogenesis and fat browning have been implicated as important mediators of wasting in animal models of acute cancer cachexia^24–26^. However, no sustained differences in body temperature (Fig. 2a) or expansion of brown adipose tissue (BAT) depots (Fig. 2b) were observed between uninfected and infected mice with *T. gondii*-induced cachexia^27^. Although transcript levels of the mitochondrial uncoupling protein *Ucp-1* were increased in the scWAT and vWAT of cachectic mice, the regulator of brown fat differentiation *Prdm16* was lower in scWAT, and other fat browning-associated transcripts, including *Pgc1a, Cidea,* and *C/ebp1* were not significantly elevated in WAT or BAT of cachectic mice (Supp. Figure 2a–c)^25^. Additionally, we did not observe the morphological changes in vWAT histology associated with fat browning (Supp. Figure 2d)^28^. These data indicate that non-shivering thermogenesis or fat browning are likely not the central drivers of sustained cachexia in *T. gondii* infection.

Consistent with other cachexia models, *T. gondii-*infected mice had small but significant reductions in fasting and non-fasting blood glucose levels (Fig. 2c,d)^29,30^. However, no significant differences in serum insulin levels were observed (Fig. 2e), and glucose clearance in response to a bolus of insulin was similar in cachectic mice and uninfected mice (Fig. 2f), suggesting that insulin resistance is not the primary driver of metabolic dysfunction during *T. gondii*-induced chronic cachexia.

**Figure 3:**
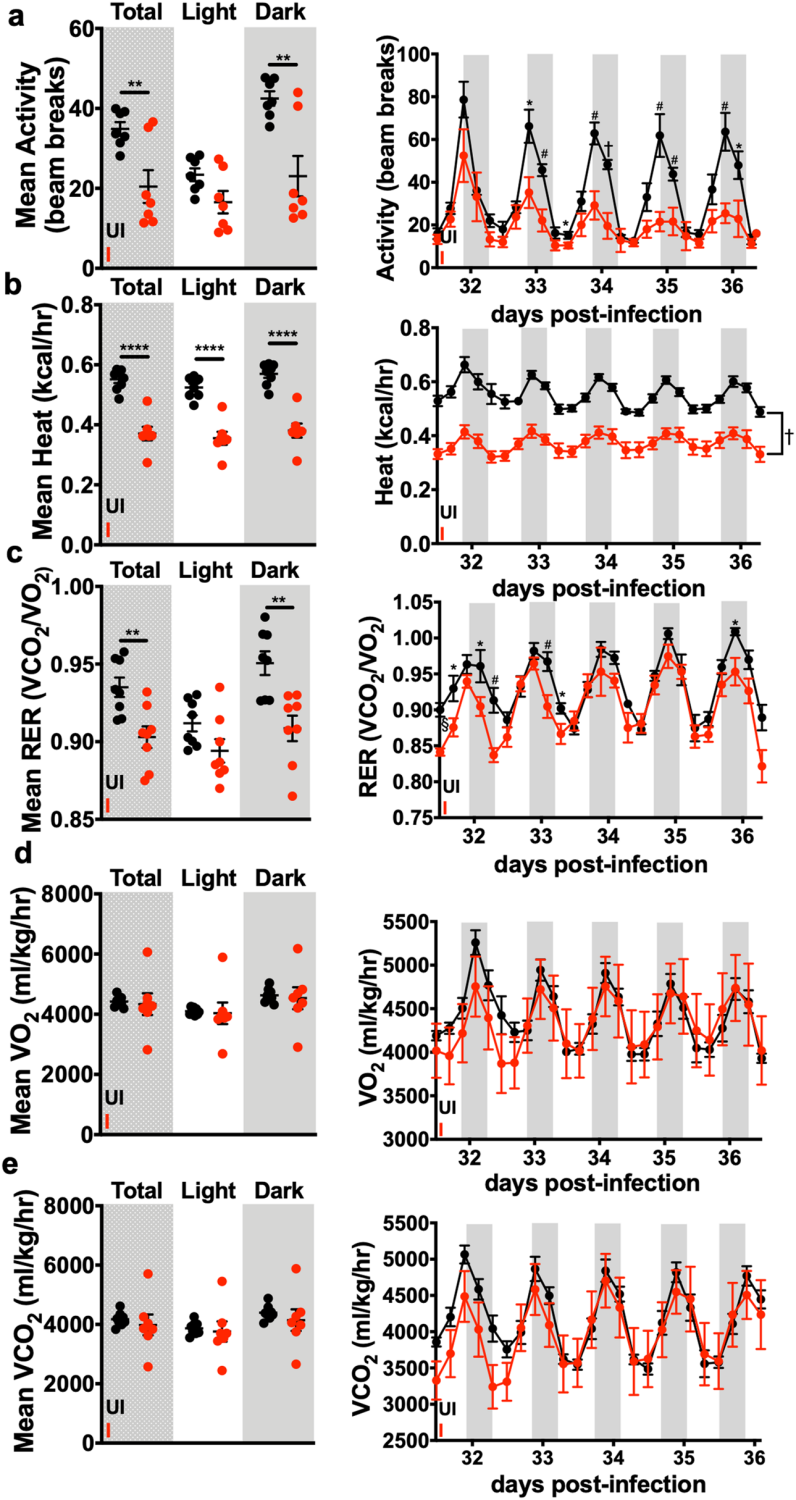
Cachectic mice have reduced activity and RER compared to uninfected mice. Mice were housed in Oxymax CLAMS metabolic cages during the time points indicated. Data from the first 24 h in the cage were excluded. Data on the left show mean of all the values from light (white bars) or dark cycles (shaded bars), or all time points combined (stippled bars). Data on the right show the 16 point rolling average of measurements with shaded bars representing the dark cycle over a 6 day span. (**a**) Activity. (**b**) Heat. (**c**) Respiratory exchange ratio (RER). (**d**) VO_2_ (normalized to lean body mass). (**e**) VCO_2_ (normalized to lean body mass). N = 7–8 animals per group, pooled between 2 independent experiments. Error bars are standard error of the mean *P < 0.05; **P < 0.01; ***P < 0.001, by unpaired Student’s *t* test (mean data) or *P < 0.05; ^#^P < 0.01; ^†^P < 0.001, ^§^P < 0.0001 (traces).

To assess systemic metabolic function during *T. gondii-*induced cachexia, mice were individually housed in Comprehensive Laboratory Animal Monitoring System (CLAMS) metabolic cages. Cachectic mice had significantly reduced nighttime activity compared to uninfected mice (Fig. 3a), as well as decreased calculated heat production (Fig. 3b), consistent with cachexia-associated fatigue. However, the reduced activity confounds our ability conclude that the reduced respiratory exchange ratio (RER) observed in cachectic mica was due to a shift towards beta-oxidative rather than glycolytic metabolism (Fig. 3c–e). To better address this question, we next measured levels of key lipolytic and metabolic signaling enzymes in vWAT, liver, and muscle by western blot. Although there was some animal-to-animal variability, no consistent differences were observed between uninfected and cachectic mice in the levels of hormone sensitive lipase (HSL), phospho-HSL (Ser660), AKT, phospho-Akt (Ser473), phospho-ACC, adipose triglyceride lipase (ATGL), phospho-ATGL, and perilipin in the WAT (Supp. Fig. 3a,b), muscle, or liver (Supp. Fig. 3c). Atglistatin, a pharmacological inhibitor of ATGL, has been shown to block muscle wasting in acute murine cancer cachexia^31^; however, it inhibits *T. gondii* growth^32^ and could not be used to confirm the conclusion that increased lipolysis was not the major driver of chronic *T. gondii*-induced cachexia. Together, these data are consistent with the observation that scWAT weight rebounds and vWAT weight stabilizes during chronic cachexia (Fig. 1f,g), clinical observations that cachexia can co-occur with obesity, and the observation that not all cachectic patients present with adipose tissue loss^1^.

### *T. gondii*-induced cachexia is associated perivascular fibrosis in metabolic organs

**Figure 4:**
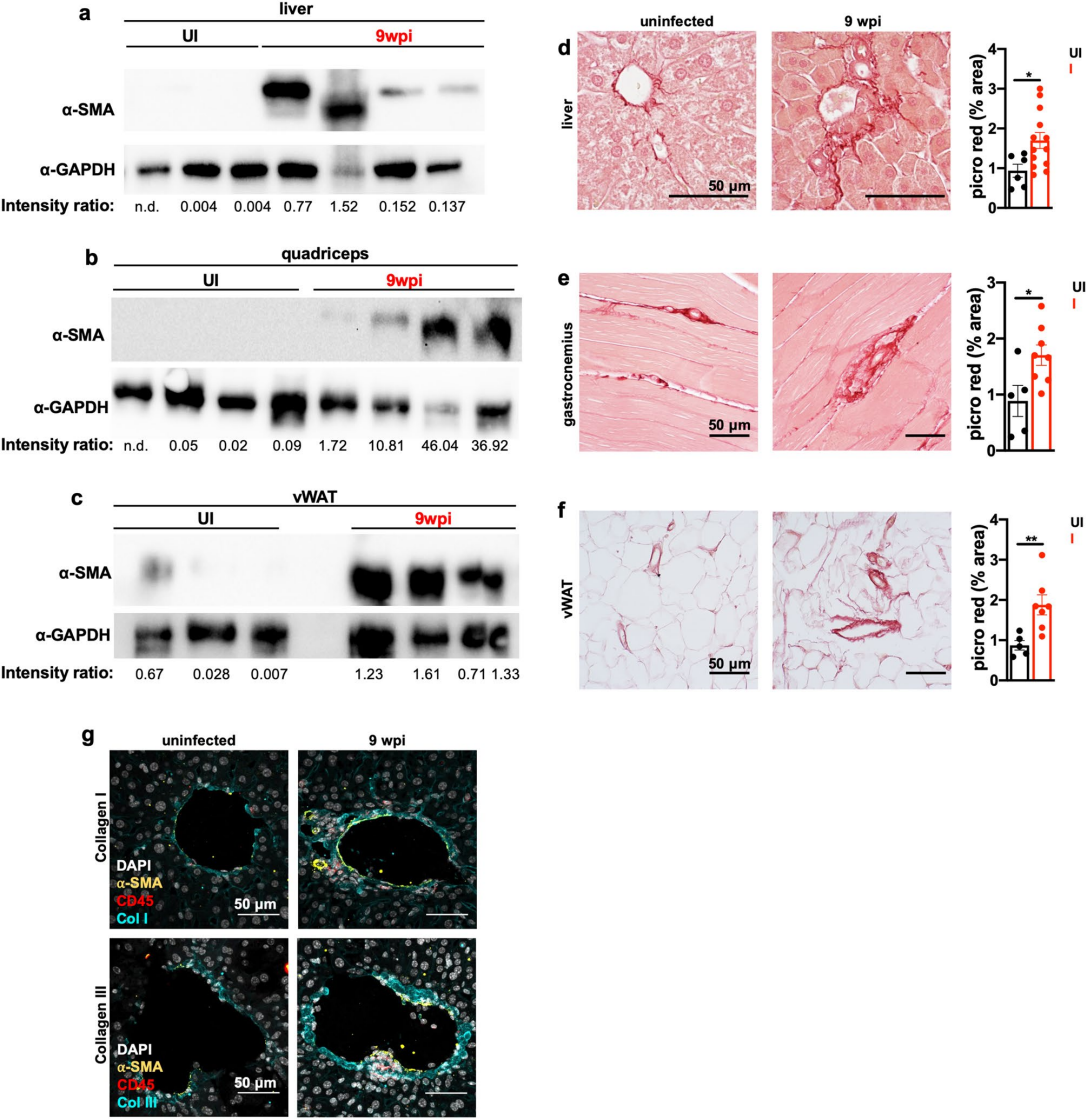
*T. gondii*-induced cachexia is associated with perivascular fibrosis in metabolic organs. (**a**–**c**) Western blot for alpha smooth muscle actin (⍺-SMA) or GAPDH on liver (**a**), quadriceps (**b**), and vWAT (**c**) lysate at 9 wpi. Representative of at least 3 independent experiments. Each lane represents an individual mouse. The intensity ratio of ⍺-SMA to GAPDH is listed below each set of bands. *n.d. *not detectable. These images were cropped from full-length blots, which are presented in Supplementary Fig. 4. (**d**–**f**) Picrosirius red staining on formalin-fixed paraffin-embedded liver (**d**), gastrocnemius (**e**), or vWAT (**f**) at 9 weeks post-infection. Representative images shown at left. Picrosirius red staining was quantified in ImageJ (right) and pixel density represented as % of each field of view. Each point represents 5 blinded fields of view averaged per mouse. (**d**) N = 6–13 mice per group, pooled between two independent experiments. (**e,f**) N = 5–8 mice per group, pooled between two independent experiments. Scale bars represent 50 µm. Error bars are standard error of the mean. *P < 0.05; **P < 0.01; ***P < 0.001 by unpaired Student’s *t* test. (**g**) Livers were harvested at 9 wpi, formalin-fixed, and then cryosectioned and stained for markers of inflammation and liver fibrosis. Maximum intensity projections of 12–17 μm thick liver sections with immunofluorescently labeled nuclei (DAPI white) ⍺-smooth muscle actin (yellow), CD45 (red) and Collagen1⍺ (top) or Collagen III (bottom) (blue) in the liver of uninfected or 9 wpi WT mice. Scale bars represent 50 μm.

In the course of probing protein expression by western blot, we noticed that the β-actin loading control was significantly upregulated in infected mice relative to uninfected controls in all the tissues assessed (Supp. Fig. 3a–c). β-Actin has over 90% sequence homology with alpha smooth muscle actin (α-SMA), and most commercially available antibodies raised against β-actin cross-react with α-SMA^33^. α-SMA is a marker of myofibroblast activation, which occurs when myofibroblast precursors (usually stromal cells with distinct homeostatic roles during quiescence) encounter local inflammation or mechanical stress. Upon activation, myofibroblasts upregulate α-SMA and deposit extracellular matrix. While this is an important aspect of wound healing, a dysregulated myofibroblast response can lead to fibrosis and impaired tissue function^34–36^. To determine if α-SMA was chronically elevated in cachectic metabolic tissues, tissue lysates were probed with an α-SMA-specific antibody by western blot. α-SMA was elevated in liver (Fig. 4a), muscle (Fig. 4b), and vWAT (Fig. 4c) of infected, cachectic mice relative to the uninfected mice at 9 weeks post-infection (full-length blots available in Supp. Fig. 4a–c). To determine if tissues with elevated α-SMA were fibrotic, tissue sections were stained with Picrosirius Red, an anionic dye that labels elongated collagen fibers. Significantly more perivascular collagen was observed in the liver (Fig. 4d), muscle (Fig. 4e), and vWAT of cachectic mice relative to uninfected controls (Fig. 4f). Increased levels of collagen I and collagen III in the liver in proximity to α-SMA-expressing cells was confirmed by immunofluorescence staining (Fig. 4g). These data indicate that perivascular fibrosis in major metabolic tissues occurs during *T. gondii* infection-induced chronic cachexia.

### IL-1α and IL-1R are expressed in the fibrotic liver microenvironment

**Figure 5:**
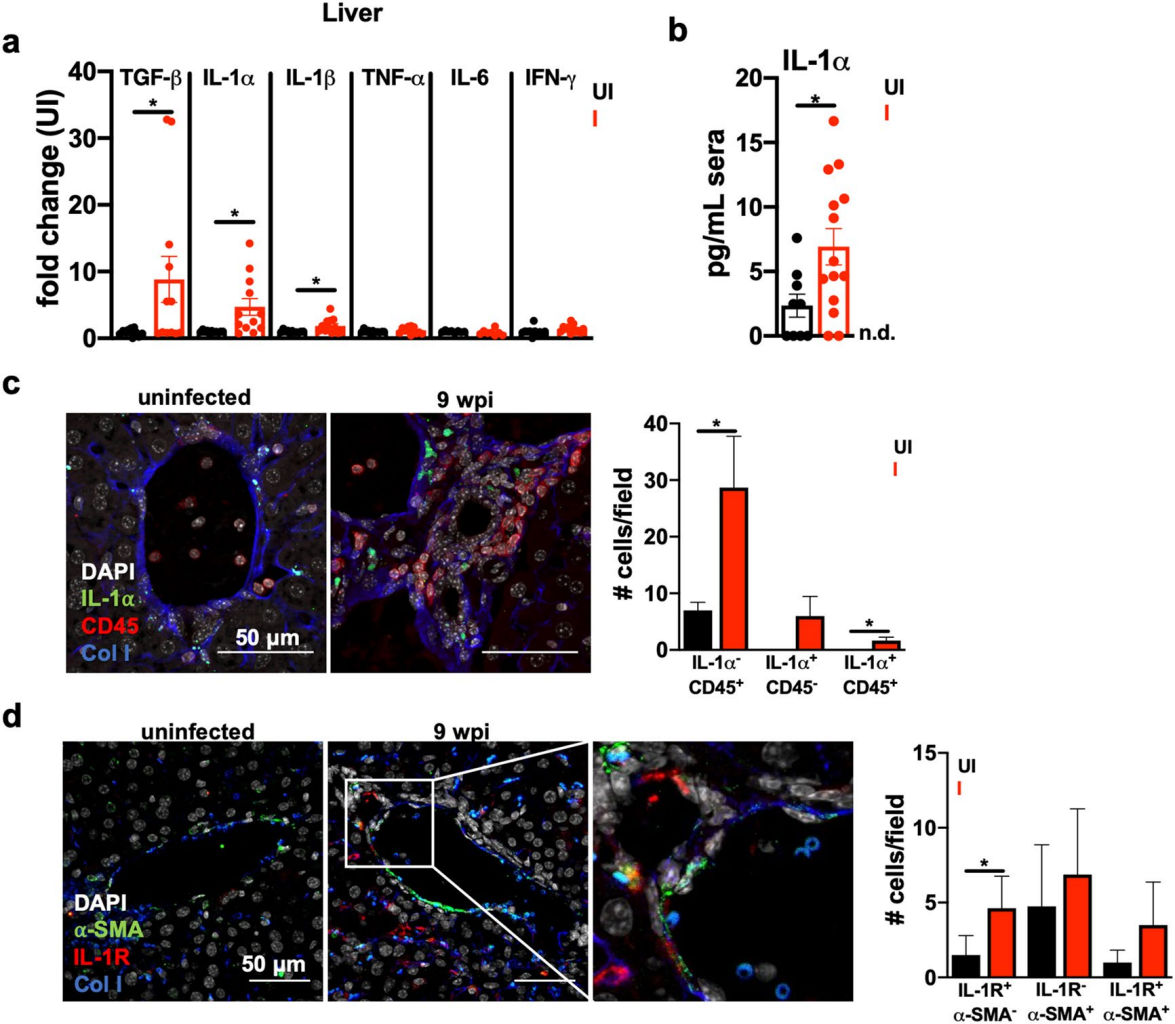
Cells expressing IL-1⍺ and IL-1R are observed in the fibrotic liver environment. (**a**) Cytokines in tissue lysates from mice at 9 wpi were measured by ELISA in liver. Data are presented as fold change relative to the mean of uninfected levels. N = 11–12 mice per group, pooled from three independent experiments. (**b**) IL-1α levels in the sera at 9 wpi measured by ELISA. N = 9–14 mice per group, pooled from four independent experiments. (**c**) Immunofluorescence labeling of nuclei (DAPI white), IL-1⍺ (green), CD45 (red), and collagen1⍺1 (blue) in the liver of UI or 9 wpi WT mice**.** Number of cells staining positive for CD45 and/or IL-1⍺, average 2–3 fields of view where immune infiltrate was present from 3 mice per  condition are quantified on the right. Error bars are standard deviation. (**d**) Immunofluorescent labeling of nuclei (DAPI white), ⍺-smooth muscle actin (green), IL-1R (red), and collagen1⍺ (blue) in the liver of uninfected or 9 wpi WT. Inset, arrow head represents ⍺-smooth muscle actin/IL-1R co-staining cells (arrow heads). Number of cells staining positive for IL-1R and/or ⍺-SMA, average of 4–8 fields of view from 3 mice are quantified on the right. Error bars are standard deviation. (**c**,**d**) represent maximum intensity projections of 9–13 μm thick z-stacks. Scale bar represents 50 μm. Error bars are standard error of the mean except where noted otherwise. *P < 0.05; **P < 0.01; ***P < 0.001 by unpaired Student’s *t* test.

Transforming growth factor beta (TGF-β) is a canonical inducer of tissue remodeling and fibrosis, so we hypothesized that TGF-β would be elevated in fibrotic tissues. As expected, TGF-β was significantly increased in liver lysates (Fig. 5a) and was trending higher in muscle and vWAT lysates (Supp. Fig. 5a,b). We also evaluated expression of IL-6, TNF-α, IFN- γ, IL-1α, and IL-1β because these inflammatory cytokine are frequently observed in cachexia as well as fibrotic diseases^37^. We found that IL-1α was significantly higher in liver lysates (Fig. 5a) and the serum (Fig. 5b) of cachectic mice at 9 weeks post-infection relative to uninfected controls. Of note, IL-6, TNF-α, and IFN-γ, which are also elevated in sera during chronic *T. gondii* infection (Fig. 1h), play a well-established and essential role in controlling chronic *T. gondii* burden. The role of IL-1α/IL-1R axis in *T. gondii* infection is comparatively understudied. IL-1 has been implicated in the development of liver fibrosis^38–40^; and altered liver biology, which is central to systemic metabolism, has been observed in a number of experimental and clinical cachexia models^41–44^. To determine if IL-1α was localized to areas of fibrosis, liver sections were stained with an IL-1α-specific antibody for immunofluorescence assays. Cells expressing IL-1α were observed within collagen I-rich perivascular fibrotic lesions, but the majority of IL-1α positive cells did not co-stain with immune cell marker CD45, suggesting that these were likely liver-resident cells (Fig. 5c). IL-1α signals through the type I IL-1 receptor (IL-1R). IL-1R staining was also observed within perivascular fibrotic lesions on a subset of α-SMA positive cells with fibroblast morphology (Fig. 5d), suggesting that α-SMA positive cells could directly respond to locally-released IL-1α in the liver.

### IL-1 is sufficient to stimulate myofibroblast contractility in vitro

**Figure 6:**
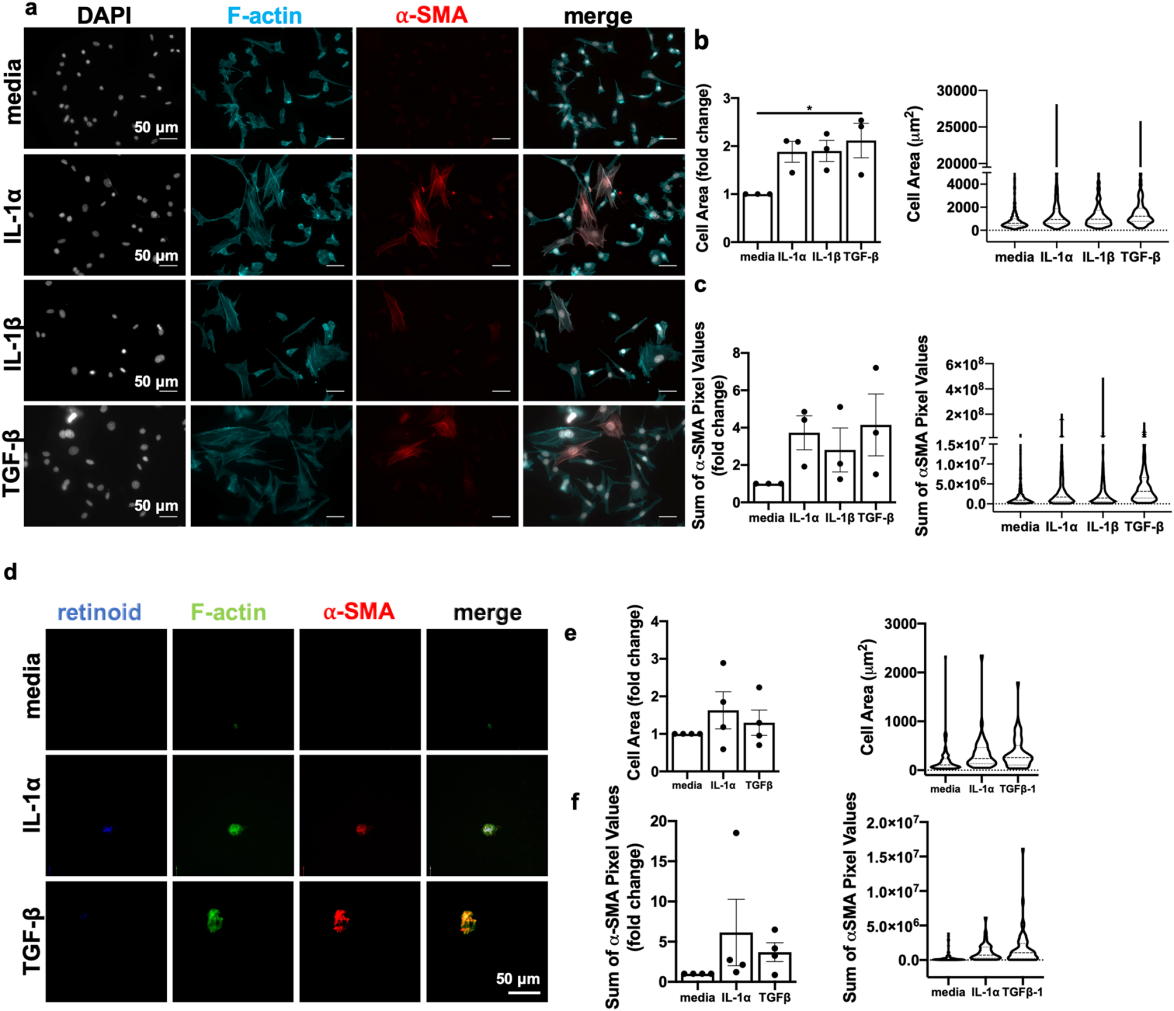
IL-1 induces contractility and alpha smooth muscle actin expression in murine embryonic fibroblasts and primary hepatic stellate cells. (**a**–**c**) MEFs were incubated with media, 10 ng/mL IL-1α, 150 pg/mL IL-1β or 10 ng/mL TGFβ-1 for 48 h. After fixation, MEFs were stained for F-actin, and alpha-smooth muscle actin (α-SMA). Cell spreading was quantified in (**b**), and levels of α-SMA expression were quantified in (**c**) as the mean relative to untreated for each biological replicate (left panels) or the single cell data pooled from three biological replicate experiments (right panels). For each experiment, 50–200 cells/group were analyzed. (**d**–**f**) Primary hepatic stellate cells (HSCs) were isolated from uninfected mouse livers, and FACS-sorted based on endogenous retinoid fluorescence (**d**). (**e**,**f**) HSCs were seeded onto 4 kPa hydrogels coated with 10 μg/mL of fibronectin and cultured with 10 ng/mL IL-1α, 10 ng/mL TGF-β, or media alone for 48 h and then fixed and stained for F-actin and α-SMA and imaged by confocal microscopy. Scale bar represents 50 μm. Total cell area quantified in (**e**) and levels of α-SMA expression were quantified in terms of pixels/cell in (**f**) as the mean relative to untreated for each biological replicate (left panels) or the single cell data pooled from four biological replicate experiments (right panels). Error bars are standard error of the mean. For the left panels, data were compared by one-way ANOVA with Bonferroni’s multiple comparisons test.

To determine if IL-1α was sufficient to promote myofibroblast activation in vitro, murine embryonic fibroblasts (MEFs) were treated with media, IL-1α, IL-1β, or TGF-β (positive control) for 48 h then stained for immunofluorescent imaging (Fig. 6a). Compared to media alone, IL-1α treatment induced α-SMA expression and increased cell surface area (a measure of increased cell contractility) to a similar level as TGF-β (Fig. 6b,c) although these were not significant when accounting for multiple comparisons analysis^45^. IL-1β treatment also increased cell spreading. By contrast, neither IL-1α or IL-1β promoted MEF proliferation or survival under serum starvation conditions (Supp. Fig. 6a).

To determine if IL-1α was sufficient to induce myofibroblast differentiation in primary cells, we took advantage of a recent protocol to isolate primary hepatic stellate cells (HSCs), the major liver myofibroblast precursor cell type in the liver, based on the endogenous autofluorescence of vitamin A-containing granules (Supp. Fig. 6b)^46^. Mechanosensing of rigid tissue culture plastic can spontaneously trigger HSC activation^47^, potentially masking an activating effect of IL-1α, so HSCs were plated on a 4kPA hydrogel to approximate normal liver rigidity^48^. IL-1α or TGF-β stimulation for 48 h led to a trend in increased cell area (Fig. 6d,e left panel) and intracellular α-SMA staining (Fig. 6f) relative to HSCs treated with media alone when single cell measurements were averaged within a biological (mouse) replicate. However, liver cell functions are specialized by lobe, zone, and proximity to arteriolar or venous blood, indicating that heterogeneity in HSC responsiveness may be expected. When single cell data was pooled across replicates, we found that response to IL-1α and TGF-β was bi-modal and that there was an increase in cell area and α-SMA staining, suggesting that at least some of the primary HSCs could be activated in response to IL-1α (Fig. 6e,f, right panels). HSCs stimulated with IL-1β for 24 h also had elevated cell area, and trended towards an increase in α-SMA which was not significant over this shorter time frame (Supp. Fig. 6c). Taken together, these data indicate that IL-1α is sufficient to promote myofibroblast activation in vitro.

### IL-1R is necessary for cachexia, as well as cachexia-associated liver and skeletal muscle atrophy and fibrosis during *T. gondii-*induced chronic cachexia

**Figure 7:**
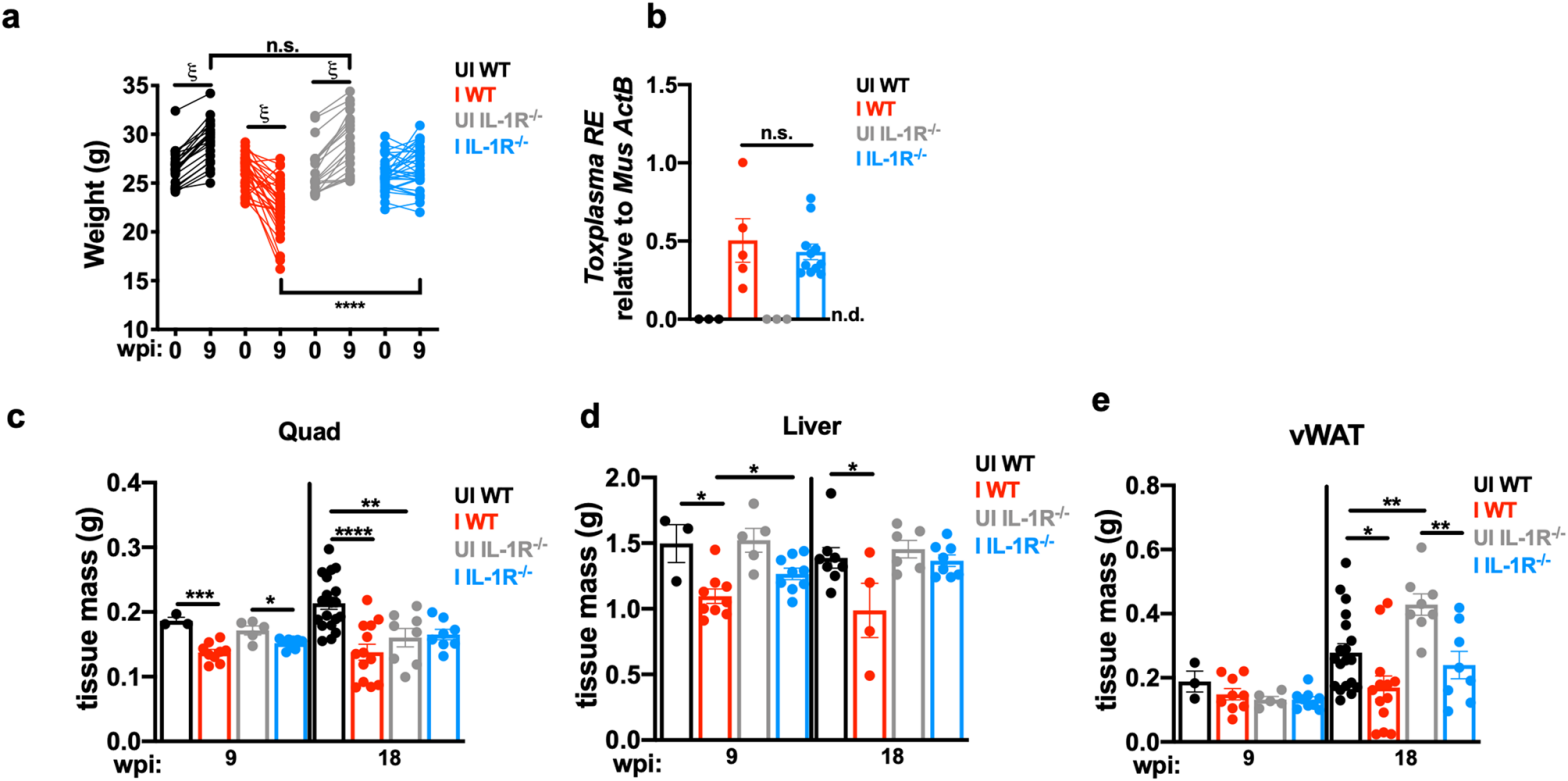
IL-1R^−/−^ mice are protected from *T. gondii*-induced cachexia and cachexia-associated liver atrophy. Total body weight of mice pre-infection and 9 wpi. N = 20–33 mice per group, pooled between 7 independent experiments. ^ξ^P < 0.0001 by paired Student’s *t* test. ****P < 0.0001 by unpaired Student’s *t* test. (**b**) Quantity of *T. gondii* DNA relative to host beta-actin in the brain at 9 wpi. N = 3–11 mice per group, representative of 3 independent experiments. (**c**–**e**) Tissue weights of quad (**c**), liver (**d**), and vWAT (**e**) at 9 or 18 wpi. 9 wpi data is representative of at least 4 independent experiments, 18 wpi data is pooled between two independent experiments. *P < 0.05; **P < 0.01; ***P < 0.001, ****P < 0.0001 by unpaired Student’s *t* test with Holm–Sidak correction for multiple comparisons.

Both IL-1α and IL-1β signal through the type I IL-1 receptor (IL-1R). To determine if the IL-1R pathway was necessary for development of fibrosis during *T. gondii* infection-induced chronic cachexia, wildtype or mice deficient in IL-1R (IL-1R^−/−^) were infected and fibrotic tissues were harvested at 9 weeks post-infection. Importantly, IL-1R^−/−^ mice were rescued from long-term weight loss (Fig. 7a). Protection was not due to better parasite clearance, as IL-1R^−/−^ and wildtype mice had a comparable brain parasite load at chronic infection (Fig. 7b). This was surprising because mice deficient in IL-6^49^, TNF-α^50^, and IFN-γ^51^ succumb to parasite overgrowth early in infection. When fibrotic tissues were weighed, infected IL-1R^−/−^ mice had less pronounced muscle loss at 9 weeks post-infection than infected wildtype mice, and by 18 weeks post-infection, IL-1R^−/−^ muscle mass completely recovered while wildtype mass remained wasted compared to uninfected controls (Fig. 7c). Similarly, infected IL-1R^−/−^ livers were significantly elevated in mass compared to infected wildtype livers at both 9- and 18 weeks post-infection (Fig. 7d). However, vWAT mass remained wasted in infected IL-1R^−/−^ and wildtype mice (Fig. 7e).

**Figure 8:**
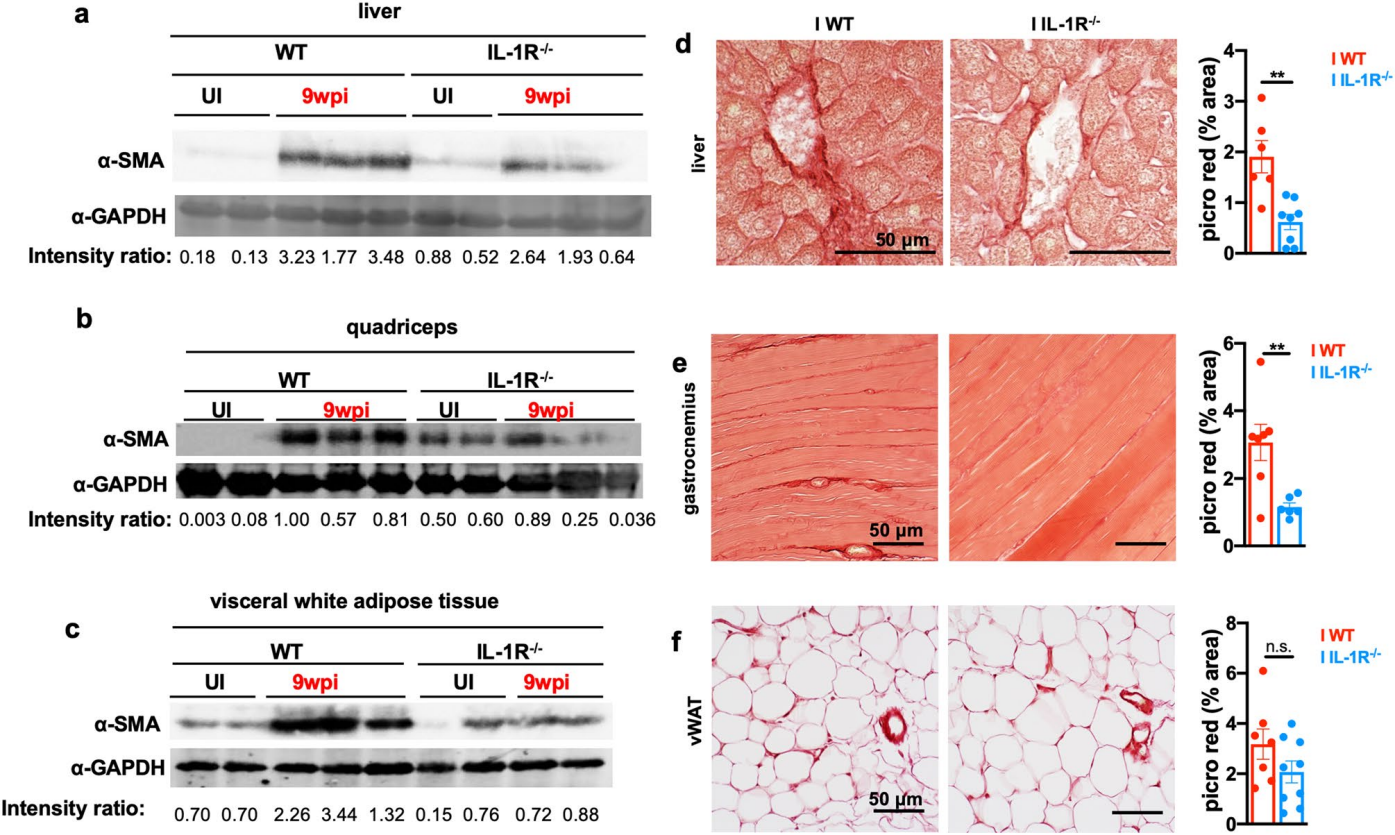
Cachectic mice develop IL-1R-dependent liver and skeletal muscle fibrosis. (**a**–**c**) Western blot for alpha smooth muscle actin (⍺-SMA) or GAPDH on liver (**a**), gastrocnemius (**b**), and vWAT (**c**) tissue lysate at 9 wpi. Representative of at least 2 independent experiments. Each lane represents an individual mouse. The intensity ratio of ⍺-SMA to GAPDH is listed below each set of bands. *n.d. *not detectable. These images were cropped from full-length blots, which are presented in Supplementary Fig. 7. (**d–f**) Picrosirius red staining on formalin-fixed tissue at 9 weeks post-infection. Picrosirius red staining was quantified in ImageJ (below) and pixel density represented as % of each field of view (each point is 5 fields of view averaged per mouse). (**d**) Liver, N = 6–8 mice per group, pooled between 2 experiments. (**e**) Gastrocnemius, N = 6–7 mice per group, pooled between 2 experiments. (**f**) vWAT. N = 6–9 mice per group, pooled between 3 experiments. Error bars are standard error of the mean. *P < 0.05; **P < 0.01; ***P < 0.001, ****P < 0.0001 by unpaired Student’s *t* test.

In comparison to WT mice, which had fibrotic tissues at 9 weeks post-infection, infected IL-1R^−/−^ mice had levels of liver α-SMA that were substantially lower than infected wildtype mice, and muscle α-SMA that was comparable to uninfected controls (Fig. 8a,b). Infected IL-1R^−/−^ vWAT, which was wasted in chronic infection (Fig. 7e), had more α-SMA than uninfected controls, but α-SMA levels were slightly lower than infected wildtype mice (Fig. 8c) (full-length blots available in Supp. Fig. 7a–c). When collagen was measured directly, infected IL-1R^−/−^ mice had significantly less picrosirius red collagen staining in the liver (Fig. 8d) and skeletal muscle (Fig. 8e) compared to infected WT mice. Although IL-1α and IL-1β were not significantly increased in skeletal muscle lysate by ELISA (Supp. Fig. 5a), the low levels of circulating IL-1α (Fig. 5b) may be sufficient to promote perivascular fibrosis in the skeletal muscle. A not mutually exclusive possibility is that pockets of IL-1α and/or IL-1β may exist in muscle fibrotic microenvironments which are below the level of detection in whole tissue lysate. We have previously demonstrated that IL-1R antagonist protein levels, which are elevated in response to IL-1R signaling, are significantly elevated in the skeletal muscle of cachectic mice, suggesting that sustained IL-1R signaling may be occurring in the muscle^14^. In the vWAT, picrosirius red staining was similar between WT and IL-1R^−/−^ mice at 9 weeks post-infection (Fig. 8f). This was consistent with vWAT wasting and α-SMA upregulation and suggest that IL-1R-independent pathways regulate collagen deposition and/or turnover in this tissue. Together, these data indicate that IL-1R drives chronic cachexia and the associated liver and skeletal muscle fibrosis during chronic *T. gondii* infection.

## Discussion

Here we show that intraperitoneal *T. gondii* infection is a robust model for chronic cachexia that recapitulates critical aspects of clinical disease. This is supported by several previous reports describing chronic weight loss and muscle dysfunction during *T. gondii* infection^13,52–55^. A major advantage of the *T. gondii* cachexia model is its longevity, which opens the door to studying mechanisms of disease reversal and testing therapeutic tools^11,56^. One outstanding question for the field is whether chronic infection is necessary to sustain cachexia. Recently, a master regulator of bradyzoite formation, Bradyzoite Factor for Differentiation 1 (BFD1), has been identified. Parasites lacking this gene have similar kinetics of acute infection but do not form cysts in the brain, suggesting that this may be a definitive tool to study the requirement for chronic infection in cachexia^57^. Our data indicate that pathways critical to acute anorexia-cachexia progression (lipolysis and non-shivering thermogenesis) may not be central drivers of chronic cachexia. Moreover, the longevity of our model allowed us to observe fibrosis in the liver, adipose tissue, and skeletal muscle, a process that typically takes many weeks to develop. This finding complements recent studies from the Seelaender group, who has recently reported increased tumor and adipose tissue fibrosis in cachectic gastrointestinal cancer patients relative to age and disease-matched, weight stable cancer patients^58,59^. Skeletal muscle fibrosis has also been reported in the quadriceps of cachectic patients with chronic heart failure^60^, as well as in the rectus abdominus of cachectic patients with pancreatic ductal adenocarcinoma (PDAC) compared to weight stable controls^61^. In the latter study, skeletal muscle collagen levels directly correlated with weight loss and mortality.

While only a handful of studies have assessed fibrosis in cachectic patients, there is a strong association between cachexia and fibrotic diseases^2,62^. Emerging clinical data show a clear correlation between muscle wasting, disease progression, and mortality in liver cirrhosis, non-alcoholic fatty liver disease, and hepatocellular carcinoma, the 4th leading world-wide cause of cancer related mortality^63–73^. The liver is a central regulator of nutrient absorption, storage, and metabolism, and as such, liver fibrosis may influence aberrant metabolism in distal tissues. Additionally, fibrosis in the skeletal muscle physically restricts muscle regeneration^74^ and fibrosis in other muscles like the heart and diaphragm could lead to dysfunction that is ultimately fatal. Historically, peripheral fibrosis during cachexia may have been overlooked because of the difficulty in obtaining biopsies from these tissues. However, muscle biopsy and assessment of adipose tissue removed during surgery are becoming increasingly more common, which may enable a more thorough understanding of cachexia-associated fibrosis in the future.

Our experiments showed that IL-1R deficient mice are protected from developing cachexia. A historical evaluation of the literature reveals that IL-1R signaling can directly induce muscle wasting; however, many of these studies were conducted before the modern, clinical definition of cachexia was established. Peripheral and central nervous system administration of IL-1 is sufficient to acutely recapitulate aspects of muscle wasting in vivo^75,76^, and in vitro, IL-1 treatment of cultured myotubes induces MuRF1 and atrogin1, the E3 ubiquitin ligases primarily responsible for muscle catabolism^77,78^. Other studies have shown that NF-κB, which is downstream of IL-1R and Toll-like receptor (TLR) signaling, is both necessary^79,80^ and sufficient to cause muscle catabolism; and constitutive NF-κB activation in skeletal muscle recapitulates a muscle wasting phenotype^81^. TLR4, which upregulates IL-1, has been shown to control muscle wasting during Lewis lung carcinoma-induced cachexia^82^; and mice deficient in MyD88, the signaling adaptor downstream of IL-1R and most TLRs, are protected from fat and muscle loss, fatigue, and mortality in a pancreatic cancer model of cachexia^83^. Our observation that IL-1R^−/−^ mice are protected from perivascular fibrosis in the liver and muscle during chronic *Toxoplasma* cachexia are consistent with these data and previous studies showing that pharmacological or genetic blockade of IL-1R signaling in mice attenuates fibrosis in the liver^38,84,85^, heart^86,87^, and lungs^87,88^. Additionally, clinical studies report that SNPs in the IL-1R antagonist gene resulting in elevated IL-1 signaling are associated with an increased risk of developing both respiratory^89,90^ and liver fibrosis^91^, suggesting that IL-1R driven fibrosis may be conserved between mice and humans. In our model, low levels of IL-1α are sustained in circulation and IL-1α-expressing cell types were observed within perivascular fibrotic lesions in the liver. This observation, in conjunction with the observation that IL-1R is also expressed within fibrotic regions, suggests that local IL-1α is sufficient to control tissue-wide development of fibrosis in the liver. Although we did not see IL-1α or IL-1β elevated in skeletal muscle lysate, we have previously shown IL-1R antagonist (which is regulated downstream of IL-1R signaling) levels to be elevated in the skeletal muscle, suggesting that IL-1R in the muscle may be responding to circulating IL-1α^14^. As an alarmin, bioactive IL-1α can be released via a broad range of cell damage or death inducers, and future work will be necessary to determine the source and mechanism of release of IL-1α during cachexia. Studies are also underway to identify precise myofibroblast cell or precursor populations that express IL-1R and test whether selectively eliminating or driving IL-1R signaling on these cell types is sufficient to influence fibrosis and cachexia during *T. gondii* infection.

There are several possible explanations for why IL-1R^−/−^ mice are not protected from adipose tissue fibrosis. First, we have previously observed that during acute *T. gondii* infection, IL-1R^−/−^ mice have more visceral white adipose tissue pathology compared to infected wildtype mice^14^. It is possible that the tissue damage incurred during acute infection is sufficient to induce IL-1R-independent fibrosis. Second, “myofibroblast” is a general term that describes heterogenous populations of cells based on ⍺-SMA expression and collagen production. Lineage tracing experiments have identified diverse myofibroblast precursor cell types, and myofibroblast gene expression and cell signaling is highly dependent on tissue residency^92^. It is possible that other inflammatory signals chronically elevated in *Toxoplasma* infection induced cachexia drive IL-1R independent myofibroblast activity in the fat. Finally, IL-1⍺ and IL-1β remain elevated in the brain during chronic *T. gondii* infection. We have not ruled out the involvement of central nervous system IL-1 signaling in the regulation of peripheral tissue homeostasis^14^.

Like cachexia, fibrosis has been notoriously difficult to target and reverse in the clinic. Our data suggest that these conditions may be linked, which poses a potential explanation for why cachexia has been so difficult to reverse with nutritional supplementation and anabolic steroids. Additional experiments will be necessary to determine if fibrosis is causal for cachexia and/or limits recovery from acute cachectic weight loss, although a recent study found that experimental models of cirrhosis lead to muscle wasting^93^. The relationship between IL-1R signaling, fibrosis and cachexia is an exciting new area for exploration in both animal models and clinical cachexia.

## Materials and methods

### Infections

To generate cysts, 8–10 week female CBA/J mice were infected with 3–10 Me49 or Me49 stably expressing green fluorescent protein and luciferase (Me49-GFP-luciferase) bradyzoite cysts by intraperitoneal injection. 4–8 weeks following infection, mice were euthanized with CO_2_ and brains were harvested, homogenized through a 70 μm filter, washed 3 times in PBS, stained with dolichos biflorus agglutinin conjugated to either FITC or rhodamine (Vector labs) and the number of cysts were determined by counting FITC-positive cysts at 20 × magnification using an EVOS FL imaging system (Thermo Fisher). 10–14 week male mice (unless otherwise noted) were infected with 10 Me49 or Me49-GFP-luciferase bradyzoite cysts by intraperitoneal infection. Prior to infection, mice were cross-housed on dirty bedding for 2 weeks to normalize commensal microbiota. Brain and tibialis anterior DNA was isolated as described^94^, and used at 100 ng DNA per qPCR reaction. For PCR of the 529 bp Repeat Element (RE), the following Taqman primer/probes were used *forward:* 5′-CACAGAAGGGACAGAAGTCGAA-3′; *reverse:* 5′-CAGTCCTGATATCTCTCCTCCAAGA-3′; *probe:* 5′-CTACAGACGCGATGCC-3′ (IDT)^95^; Mm02619580_g (ThermoFisher Scientific) was used as the mouse beta actin reference gene.

### Mouse strains/husbandry

C57BL/6 and IL-1R^−/−^ mice were purchased from Jackson Laboratories. All mice were bred in-house. All animal protocols were approved by the University of Virginia Institutional Animal Care and Use Committee (protocol # 4107-12-18). All animals were housed and treated in accordance with AAALAC and IACUC guidelines at the University of Virginia Veterinary Service Center.

### Sorting and culturing of primary hepatic stellate cells

Primary murine hepatic stellate cells were isolated as previously described^46^. Following sorting on a BD Influx Cell Sorter in the University of Virginia Flow Cytometry Core, cells were seeded onto fibronectin coated 4 kPa polyacrylamide hydrogels (Matrigen) and stimulated with 10 ng/mL recombinant mouse IL-1α (R&D), 10 ng/mL recombinant mouse TGF-β (R&D), or media alone for 48 h, after which hydrogels were fixed with 4% paraformaldehyde and stained with phalloidin-488 (Invitrogen) and anti α-SMA antibody (Invitrogen, clone IA4). Cells were mounted with ProLong Diamond Anti-Fade mountant (Thermo Fisher Scientific), and imaged at room temperature on a Nikon Eclipse Ti microscope with an UltraView VoX imaging system (PerkinElmer) using a Nikon N Apo LWD 40 × water objective (numerical aperture: 1.15) and cell area and α-SMA intensity were determined using Volocity software.

### Mouse embryonic fibroblasts

Transformed mouse embryonic fibroblasts were cultured (DMEM, 10% FBS, 1% l-glutamine, 1% penicillin/streptomycin, 1% HEPES, 1% sodium pyruvate) and used between passage 3–10. 1 × 10^4^ cells were seeded overnight onto poly-D-lysine coated glass coverslips in 24-well plates and then stimulated with 10 ng/mL recombinant mouse IL-1α (R&D), 10 ng/mL recombinant mouse TGF-β (R&D), or media alone for 48 h. Coverslips were fixed in 4% paraformaldehyde for 10 min, permeabilized with 0.1% Triton X-100 for 15 min, blocked in 1% BSA for 30 min, and then stained overnight at 4 °C with anti α-SMA antibody (Invitrogen, clone IA4). The next day, coverslips were stained for 1 h at room temperature with phalloidin-eFluor660 (eBioscience) or donkey anti-mouse-AF594 (Jackson ImmunoResearch), and mounted onto slides with Vectashield Mounting Medium containing DAPI (Vector Laboratories). Coverslips were imaged on a Zeiss Imager M2 microscope (Carl Zeiss) with an AxioCam Mrm camera (Carl Zeiss) using a 20 × objective (numerical aperture: 0.80) and ZenBlue software (Carl Zeiss). Cell area and α-SMA was determined by defining each cell as a region of interest by tracing each cell in Fiji software and quantifying cell area and α-SMA intensity. For serum starvation experiments, 2500 MEFs/well were seeded into a 96 well plate overnight and then treated for 48 h with cytokine in media containing either 10% or 1% serum. At the end of the 48-h period, cell proliferation and viability were determined using CellTiter-Glo reagent (Promega).

### Body temperature measurements

Mice were anesthetized with isoflurane at 10 days post-infection, and subcutaneously injected with temperature micro-transponders (Bio Medic Data Systems, Seaford, DE). Temperature was monitored daily (at the same time of day) using a telemetric reader.

### Bomb calorimetry

Mice were individually housed for 24 h at 10 weeks post-infection. Fecal pellets were collected every 2 h during the daytime and flash-frozen. Pellets were lyophilized and then sent to University of Texas Southwestern Metabolic Phenotyping Core for bomb calorimetric analysis.

### CLAMS metabolic monitoring

At 5 weeks post-infection, mice were individually housed in Oxymax cages for 5 days (CLAMS, Columbus Instruments, Columbus, OH) at the University of Virginia. Mice were maintained at 25 °C with 12 h light/dark cycles and had free access to food and water in all conditions. The first 24 h of data was considered and acclimation period and excluded from analysis. 16-point rolling averages across the remaining data were used to visualize data across the recorded time periods.

### Western blots

Flash-frozen tissues were homogenized by bead-beating in lysis buffer (25 mM Tris–HCl, 15 mM NaCl, 1 mM MgCl_2_, 2.7 mM KCl, 1 mM EDTA, 1 mM EGTA, 1 mM DTT, 1% Triton-X, and protease and phosphatase inhibitors (Roche EDTA-free protease inhibitor mini and Pierce Phosphatase Inhibitor Mini Tablets), and centrifuged. Protein concentration was measured using via BCA concentration (Pierce BCA Protein Assay Kit, Cat# 23225). Protein concentrations were equalized by diluting with 2 × Laemmli buffer and lysate was then boiled at 95 °C for 3 min. All protein samples were separated on 10% bis–Tris gels by SDS-PAGE and then transferred to PVDF membranes using Trans-Blot Turbo Transfer System (Biorad). The membranes were blocked in 2% ECL Prime Blocking Reagent (GE Amersham RPN418) in TBS-T for 30 min, incubated in primary antibody for 1 h at room temperature, washed 3 × in TBS-T, incubated in secondary antibody for 30 min at room temperature, washed 3 × in TBS-T and then imaged on either the Bio-Rad ChemiDoc Imager (for HRP secondary antibodies) or the Leica Typhoon (for fluorescently-conjugated secondary antibodies). The following primary antibodies were used to probe protein levels by immunoblotting: anti-GAPDH (Cell Signaling Technology, clone D16H11), anti-β-Actin (Santa Cruz Biotechnology, clone 1), anti- α-SMA (Santa Cruz Biotechnology, clone CGA7), anti-pACCα (Santa Cruz Biotechnology, clone F-2), anti-ATGL (Santa Cruz Biotechnology, clone F-7), anti-HSL (Cell Signaling Technology, #4107), anti-phospho-HSL (Ser660) (Cell Signaling Technology, #4126), anti-AKT (Cell Signaling Technology, clone C67E7), and anti-phospho-AKT (Cell Signaling Technology, clone D9E). The following secondary antibodies were used: goat anti-rabbit Cy5 (Jackson Immunoresearch, 705-175-147), goat anti-mouse HRP (Thermo Scientific, PA1-28,664). All primary antibodies were used at a 1:1000 dilution and secondary antibodies at a 1:10,000 dilution in 2% ECL Prime Blocking Reagent. The stripping protocol used was adapted from Yeung and Stanley, 2009^96^. Briefly, blots were washed 2 times with TBS-T, incubated twice for 5 min each in GnHCl stripping solution at room temperature (6 M GnHCl, 0.2% NP-40, 0.1 M β-mercaptoethanol, 20 mM Tris–HCl), washed 4 times (3 min each) in wash buffer at room temperature (0.14 M NaCl, 10 mM Tris–HCl, 0.05% NP-40), and then blocked in 2% ECL Primer Blocking Reagent in TBS-T. Stripped blots were re-probed following the protocol described.

### Cytokine measurements

Sera cytokines were measured by Luminex at the University of Virginia Flow Cytometry Core, or by ELISA (Thermo Fisher Scientific). Cytokine measurement on tissue homogenate was assessed using ELISA (Mouse IFN gamma Uncoated ELISA (Invitrogen, #88-7314); Mouse TNF alpha Uncoated ELISA (Invitrogen, #88-7324); IL-1 alpha Mouse Uncoated ELISA Kit (ThermoFisher #88-5019-88); eBioscience Mouse IL-6 ELISA Ready-SET-Go! Kit (Fisher Scientific #50-112-8863; IL-1 beta Mouse Uncoated ELISA Kit (Thermo Fisher Scientific #88-7013-88).

### qPCR on adipose tissue

Flash frozen tissues were homogenized in TRIzol reagent (Invitrogen) by bead-beating and RNA was extracted following manufacturer’s instructions. Following genomic DNA digestion and reverse transcription, RNA was run on a QuantStudio 6Flex (Applied Biosystems) using ABI Power SYBR Green PCR Master Mix (Applied Biosystems). Primers used: *ActB *(*Forward:* 5′-GGCTGTATTCCCCTCCATCG-3′, Reverse: 5′-CCAGTTGGTAACAATGCCATGT-3′), *Ucp-1 *(*Forward: 5′-ACTGCCACACCTCCAGTCATT-3′*, *Reverse: 5′-CTTTGCCTCACTCAGGATTGG-3′*), *Prdm16 *(*Forward:* 5′-GAAGTCACAGGAGGACACGG-3′, *Reverse:* 5′-CTCGCTCCTCAACACACCTC-3′), *PGC1α* (*Forward:* 5′-*ACAGCTTTCTGGGTGGATTG-*3′, *Reverse:* 5′-TGAGGACCGCTAGCAAGTTT-3′), *Cidea* (*Forward:* 5′*-*TGCTCTTCTGTATCGCCCAGT-3′*, Reverse: 5′-GCCGTGTTAAGGAATCTGCTG-*3*′*), *C/ebpβ *(*Forward:* 5′-TGACGCAACACACGTGTAACTG-3′, Reverse: 5′-AACAACCCCGCAGGAACAT-3′), *Pparγ-2* (*Forward:* 5′-TCGCTGATGCACTGCCTATG-3′, *Reverse*: 5′-GAGAGGTCCACAGAGCTGATT-3′). Data were analyzed using the ΔCt method (relative to a housekeeping gene), and then normalized to the mean of the ΔCt of uninfected mice to get fold change. Any samples that did not amplify were set to the Ct value of 40 for purposes of quantification.

### Picrosirius red

Formalin-fixed, paraffin-embedded tissue was stained with picrosirius red, and slides were imaged on an Olympus BX51 microscope with an Infinity 1 camera (Lumenera) for brightfield or a Zeiss Apotome2 (Carl Zeiss, Germany) under polarized light using the 20 × or 40 × objective. 5–10 blinded fields of view were acquired per mouse. To quantify percent area, images were binarized in Fiji^97^, thresholded, and percentage of positive pixels per area was determined.

### Immunofluorescence

Following euthanasia and harvest, tissues were fixed overnight in 4% paraformaldehyde at 4 °C, after which they were submerged in 30% sucrose in PBS, embedded in Tissue-Tek O.C.T. Compound (VWR) and flash frozen on dry ice. Samples were submitted to the Research Histology Core at the University of Virginia for sectioning.

For IL-1α staining, slides were blocked for 1 h at room temperature in blocking buffer (2% donkey serum, 2% goat serum, 0.1% Triton-X, 0.05% Tween-20). Samples were incubated overnight at 4 °C in primary antibody diluted in blocking buffer (R&D AF-400-NA goat anti-IL-1 α, 1:50; Novus Biologicals NB600-408 rabbit anti-collagen I, 1:50; Biolegend 103101 rat anti-CD45, 1:50). The next morning, samples were washed 3 times in PBS/0.1% Triton-X and were incubated in secondary antibody (donkey anti-goat Dylight 594, Novus NBP1-75607, 1:500; Invitrogen A21245 goat anti-rabbit AF647, 1:300; and donkey anti-rat AF488, 1:500), for 1 h at room temperature, diluted in blocking buffer. Samples were washed 3 times in PBS, stained for 5 min in 10 μg/mL DAPI, washed 3 times in PBS, and mounted in Vectashield. Slides were imaged on a Zeiss LSM 880 confocal microscope using the 40 × or 63 × objectives.

For IL-1R staining, antigen retrieval was performed by boiling samples in sodium citrate buffer (10 mM sodium citrate buffer + 0.05% Tween-20, pH 6.0) for 20 min and then washing 3 times in PBS. They were blocked for 1 h at room temperature in 2% donkey serum and CD16/CD32 (Biolegend, Clone 93, 1:200). Samples were incubated overnight at 4 °C in primary antibody diluted in blocking buffer (R&D AF771 goat anti-IL-1R, 1:50; Novus Biologicals NB600-408 rabbit anti-collagen 1, 1:50). The next morning, samples were washed 3 times in PBS/0.1% Triton-X and were incubated in secondary antibody (Novus NBP1-75607 donkey anti-goat Dylight 594, 1:250 and LifeTech A21206 donkey anti-rabbit AF488, 1:200), and the directly conjugated α-SMA primary (Novus Biologicals NBP2-34760APC, 1:400) for 1 h at room temperature, diluted in PBS/0.1% Triton-X. Samples were washed 3 times in PBS, stained for 5 min in 10 μg/mL DAPI, washed 3 times in PBS, and mounted in Vectashield (Vector Laboratories).

For collagen I and III staining, samples were boiled in sodium citrate buffer (10 mM sodium citrate buffer + 0.05% Tween-20, pH 6.0) for 20 min and then washed 3 times in PBS + 0.1% Triton-X. They were blocked for 1 h at room temperature in blocking buffer (2% donkey serum + 2% goat serum).

Samples were incubated overnight at 4 °C in primary antibody diluted in blocking buffer (Biolegend 103101 rat anti-CD45, 1:50; Novus Biologicals NB600-408 rabbit anti-collagen I, 1:50; Novus Biologicals NB600-594 rabbit anti-collagen III, 1:50). The next morning, samples were washed 3 times in PBS + 0.1% Triton-X and were incubated in secondary antibody (Invitrogen A-11007 goat-anti rat AF594, 1:500; LifeTech A21206 donkey anti-rabbit AF488, 1:200), and the directly conjugated α-SMA primary (Novus Biologicals NBP2-34760APC, 1:400) diluted in PBS + 0.1% Triton-X for 1 h at room temperature. Samples were washed 3 times in PBS, stained for 5 min in 10 μg/mL DAPI, washed 3 times in PBS, and mounted in Vectashield (Vector Laboratories). Slides were imaged on a Zeiss LSM 880 confocal microscope (Carl Zeiss) using the 40 × oil (numerical aperture: 0.09) or 63 × oil (numerical aperture: 0.09) objectives and ZenBlack software (Carl Zeiss).

### Statistical analysis

Statistics were performed using GraphPad Prism 8. For comparison of two groups, two-tailed unpaired Student’s *t* tests were performed with a confidence level of 95%. For comparison across multiple groups, two-tailed unpaired Student’s *t* test with Holm–Sidak correction or one-way ANOVA with Bonferroni correction for multiple comparisons was used where noted. Statistical significance threshold was set at P ≤ 0.05. All data are presented as the mean ± SEM except where noted otherwise.

## Supplementary information

Supplementary Figures.
