# Supplementary material for: T. gondii infection induces IL-1R dependent chronic cachexia and perivascular fibrosis in the liver and skeletal muscle
Source: Sci Rep. 2020 Sep 24;10:15724. doi: 10.1038/s41598-020-72767-0 (PMC7515928; doi:10.1038/s41598-020-72767-0)
Supplement: Supplementary file 1 — Supplementary Figures. [file 41598_2020_72767_MOESM1_ESM.pdf]

T. gondii infection induces IL-1R dependent chronic cachexia and perivascular fibrosis in the liver and skeletal muscle

Stephanie J. Melchor[1], Jessica A. Hatter[2], Érika A. LaTorre Castillo[3], Claire M. Saunders[1], Kari A. Byrnes[1], Imani Sanders[1], Daniel Abebayehu[4], Thomas H. Barker[4], Sarah E. Ewald[1*]

[1]Department of Microbiology, Immunology, and Cancer Biology and The Carter Immunology Center, University of Virginia School of Medicine, Charlottesville VA, USA, [2]Department of Pharmacology, University of Virginia School of Medicine, Charlottesville VA, USA, [3]Ponce Health Sciences University, Ponce PR, USA, [4]Department of Biomedical Engineering, University of Virginia School of Medicine, Charlottesville VA, USA.

* corresponding author: se2s@virginia.edu, (p) 434-924-1925, (f) 434-924-1212 ORCID: http://orcid.org/0000-0002-5327-7578

Supplementary Figure Legends

Supplementary Figure 1. Skeletal muscle is wasted in mice chronically infected with *T. gondii.* A, Daily percent weight change of 10-14 week old female C57Bl/6 mice that were intraperitoneally infected with 10 Me49-GFP-luciferase *T. gondii* cysts or mock infected. N= 5-11 mice per group. **B,** Tissue weights of gastrocnemius (GA), tibialis anterior (TA) and extensor digitorum longus (EDL) at 10 wpi. N= 7-15 mice per group, pooled between 2-3 independent experiments *P < 0.05; **P < 0.01; ***P < 0.001 by unpaired Student's T test.

Supplementary Figure 2: *Toxoplasma*-induced cachexia does not induce the transcriptional signature associated with adipose tissue beigeing at 17 weeks post-infection. 10-14 week old C57BL/6J mice (uninfected, UI and infected, I) were intraperitoneally infected with 10 Me49-GFP-luciferase Toxoplasma cysts. **A-C**, qPCR for markers of fat browning and thermogenesis in inguinal subcutaneous adipose tissue (scWAT) **(A)** epigonadal visceral white adipose tissue (vWAT) **(B)** or supraclavicular brown adipose tissue (BAT) **(C)** at 17 wpi. Error bars are standard error of the mean. N= 7-9 mice per group. *P < 0.05; **P < 0.01; ***P < 0.001 by unpaired Student's T test. **D,** H&E staining of vWAT at 9 wpi. Scale bar represents 50 µm.

Supplementary Figure 3: Lipolysis is not a main driver of *Toxoplasma*-induced cachexia. 10-14 week old C57BL/6J mice (uninfected, UI and infected, I) were intraperitoneally infected with 10 Me49-GFP-luciferase *Toxoplasma* cysts. **A-C**, Tissues were harvested at 5 wpi. Tissue lysates were made for subcutaneous white adipose tissue (scWAT) and epigonadal visceral white adipose tissue (vWAT) (**A-B**) or quadriceps muscle (QUAD) and liver (**B**), and blotted for lipolysis machinery: phosphorylated and non-phosphorylated hormone sensitive lipase (p-HSL, HSL), phosphorylated and non-phosphorylated AKT, phosphorylated (p-ACC), perilipin. Beta-actin and GAPDH were loading controls. Each lane is lysate from an individual mouse. Representative of 2 independent experiments. Full length blots shown in boxes on the right. Dashed lines indicate the cropped section.

Supplementary Figure 4: Full-length blots from Figures 4A-C. Full-length blots from Figure 4A-C corresponding to a-SMA (left) and GAPDH (right) in the liver (**A**), quadriceps (**B**), and vWAT (**C**). Dashed lines indicate the cropped section as displayed in the main body figure.

Supplementary Figure 5. IL-1β is locally elevated in cachectic skeletal muscle. A-B, Cytokines in tissue lysates from mice 9 wpi were measured by ELISA in quad **(A)** quad and vWAT **(B).** Data are presented as fold change relative to the mean of uninfected levels. N=11-12 mice per group, pooled from three independent experiments for all cytokines except TGF-β in the vWAT (N=4).

Supplementary Figure 6: IL-1 does not promote survival or proliferation in mouse endothelial fibroblasts. A, MEF cells were plated on 96 well plates in 10% normal sera or 1% normal sera and incubated with media, IL-1α, IL-1β or TGFβ-1 for 48 hours. Cell number was determined relative to a standard curve using CellTiter-Glo reagent. Data pooled between 3 experiments. $^{*}P < 0.05$; $^{**}P < 0.01$; $^{***}P < 0.001$ by unpaired Student's T test. **B-C,** Primary hepatic stellate cells (HSCs) were isolated from uninfected mouse livers, and FACS sorted based on endogenous retinoid fluorescence. HSCs were seeded onto 4kPa hydrogels coated with 10ug/mL of fibronectin and cultured with 10ng/mL of IL-1b or media alone for 24hrs and then fixed and stained for F-actin and α-SMA and imaged by confocal microscopy. Total cell area and levels of α-SMA expression were quantified in terms of pixels/cell as the mean relative to untreated for each biological replicate (left panels) or the single cell data pooled from two biological replicate experiments (right panels).

Supplementary Figure 7: Full-length blots from Figures 8A-C. Full-length blots from Figures 8A-C corresponding to a-SMA (left) and GAPDH (right) in the liver (**A**), quadriceps (**B**), and vWAT (**C**). Dashed lines indicate the cropped section as displayed in the main body figure.

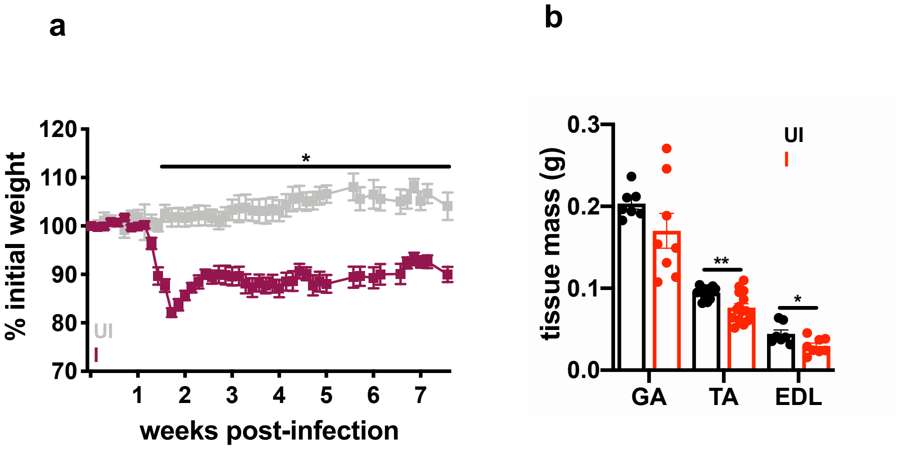

Supplementary Figure 1

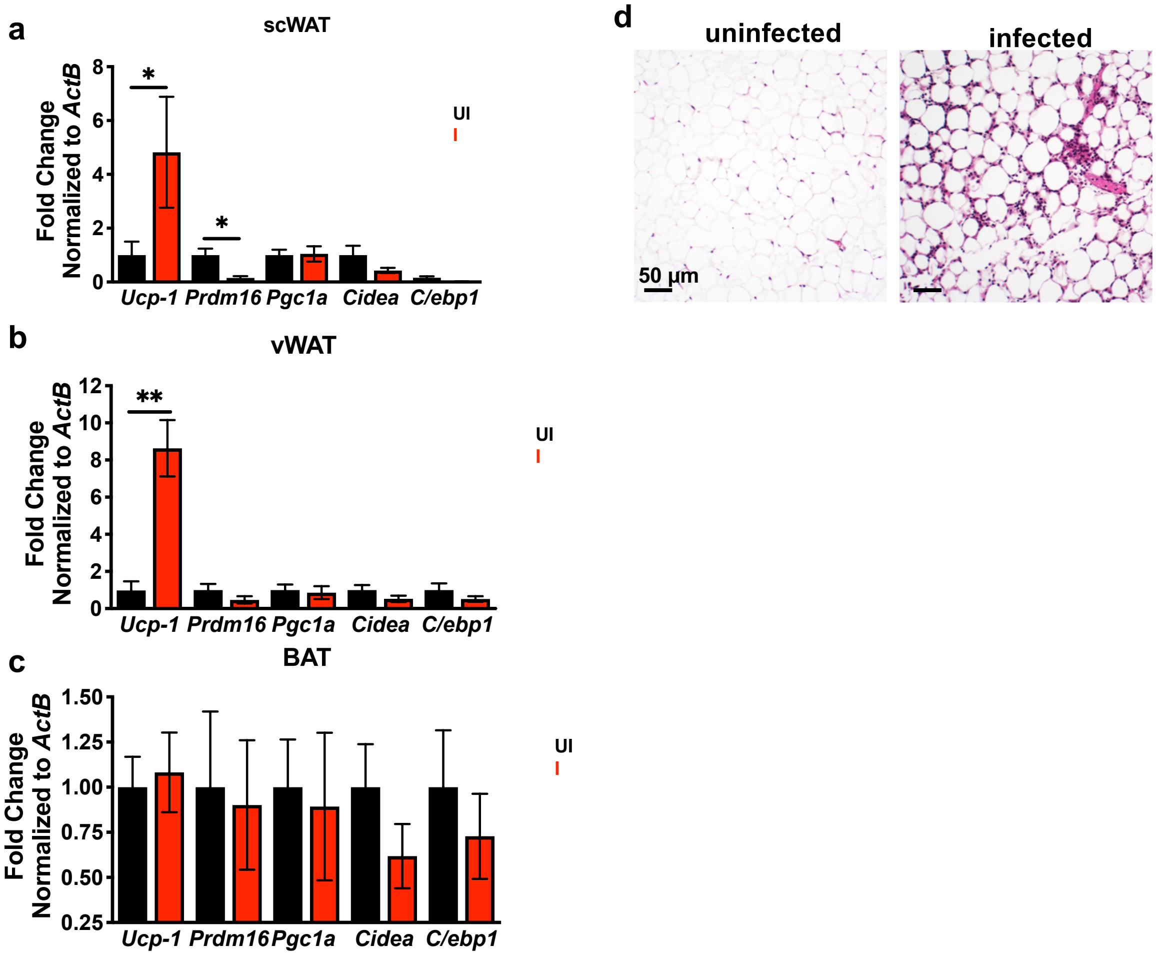

Supplementary Figure 2

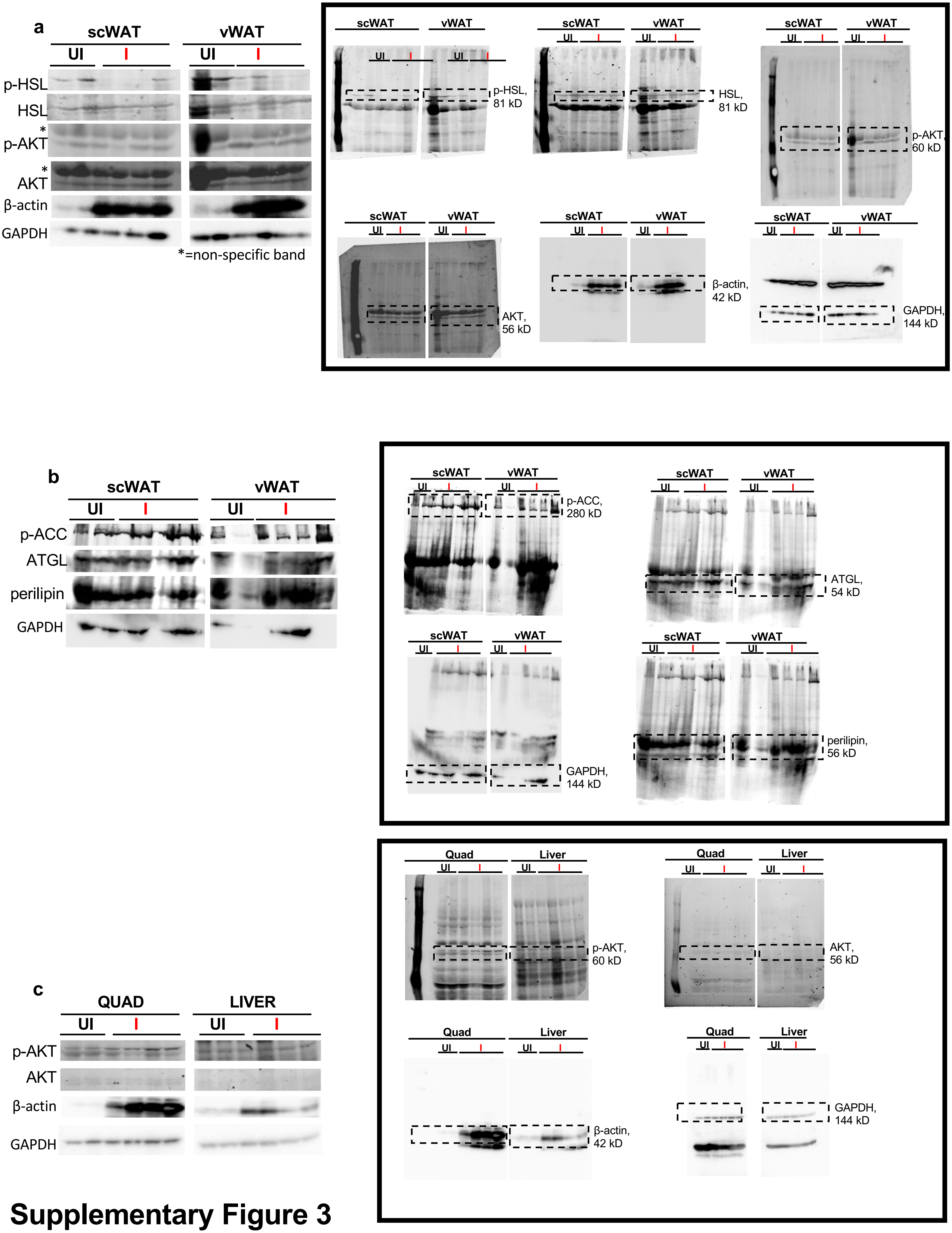

Supplementary Figure 3

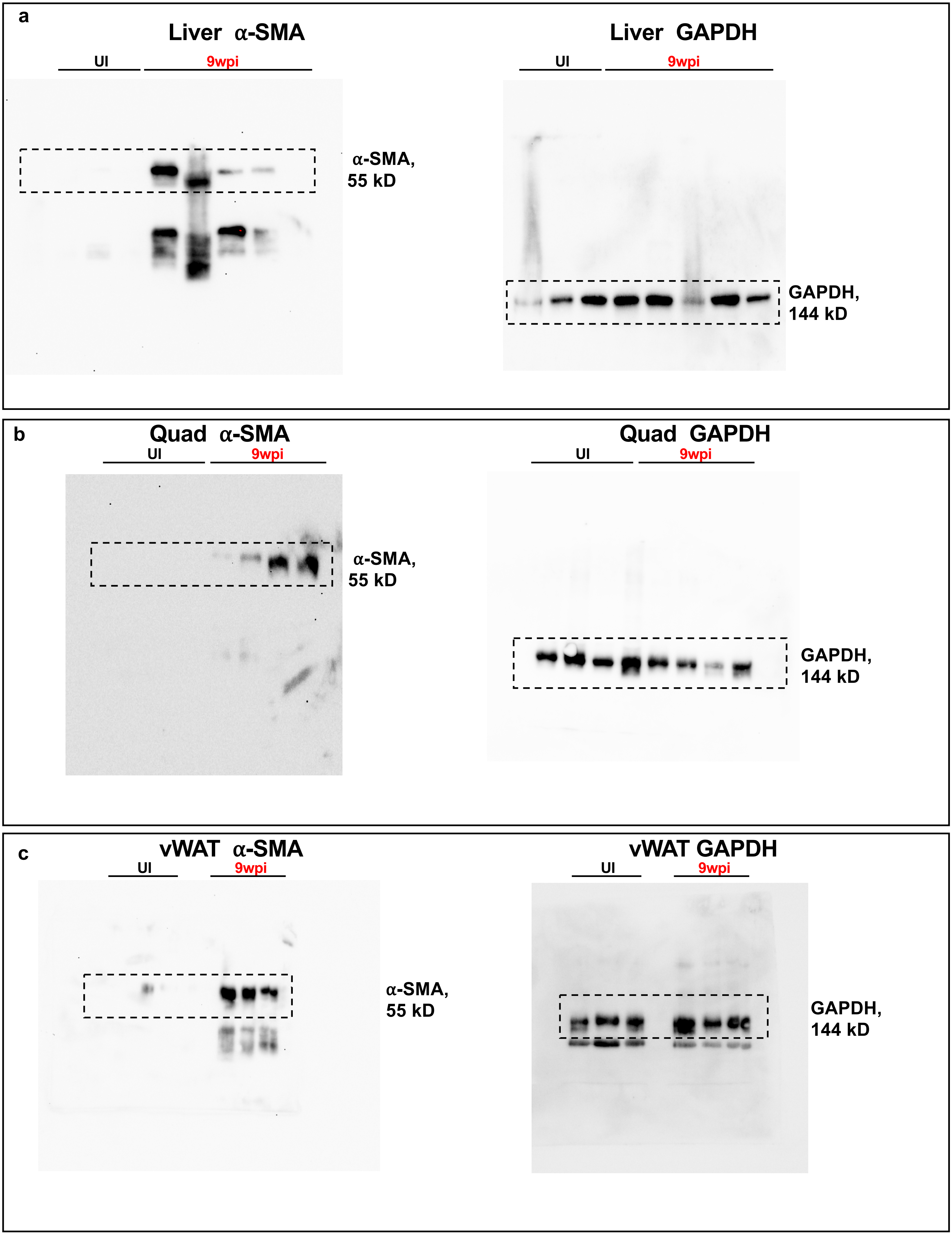

Supplementary Figure 4

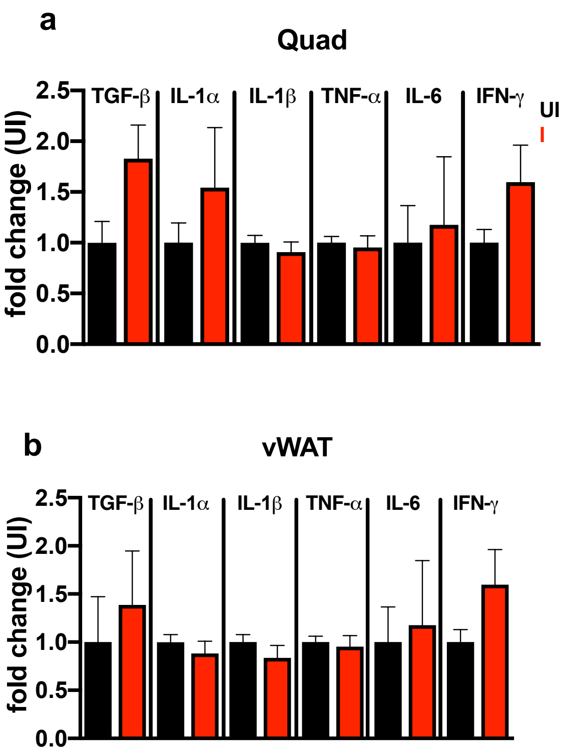

Supplementary Figure 5

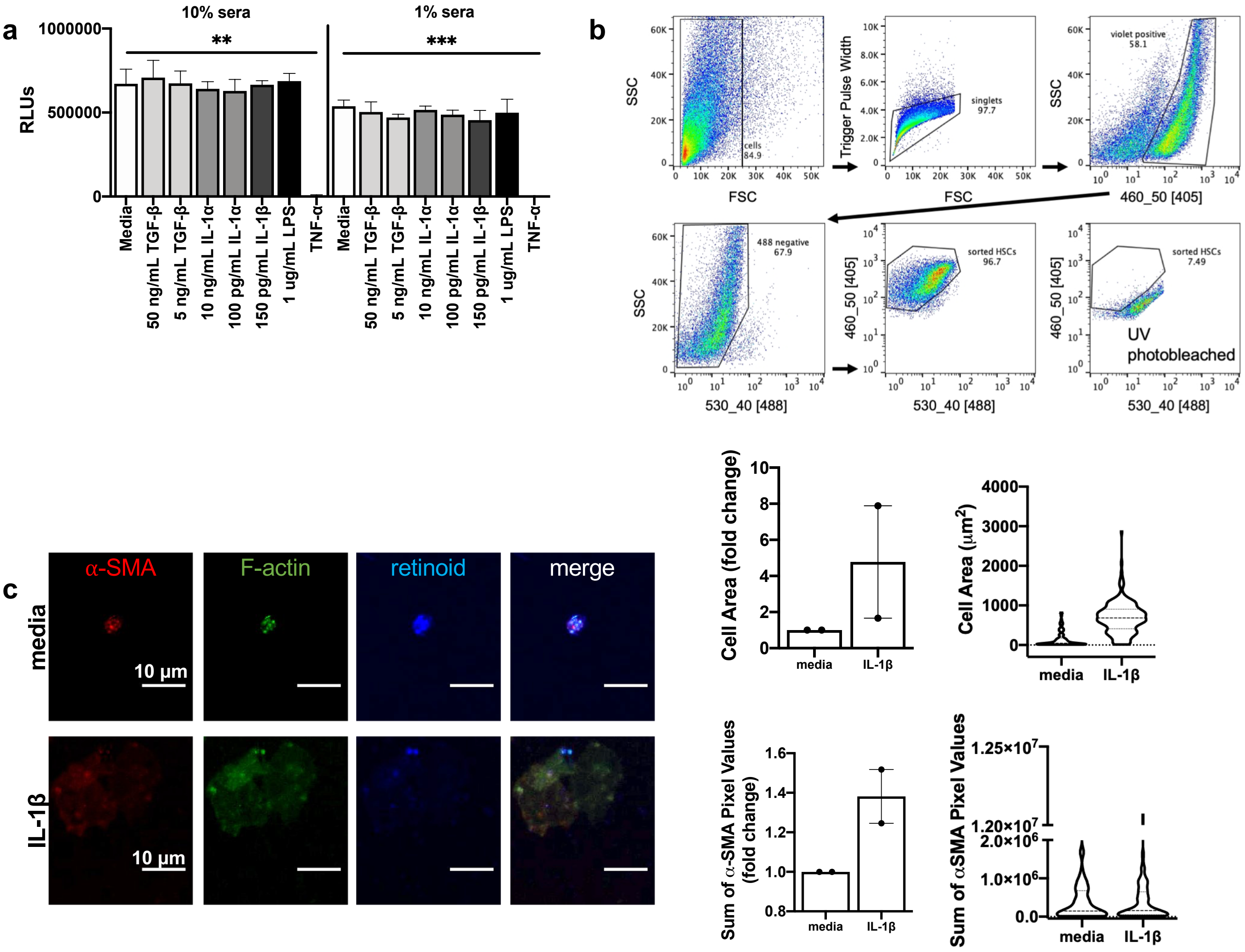

Supplementary Figure 6

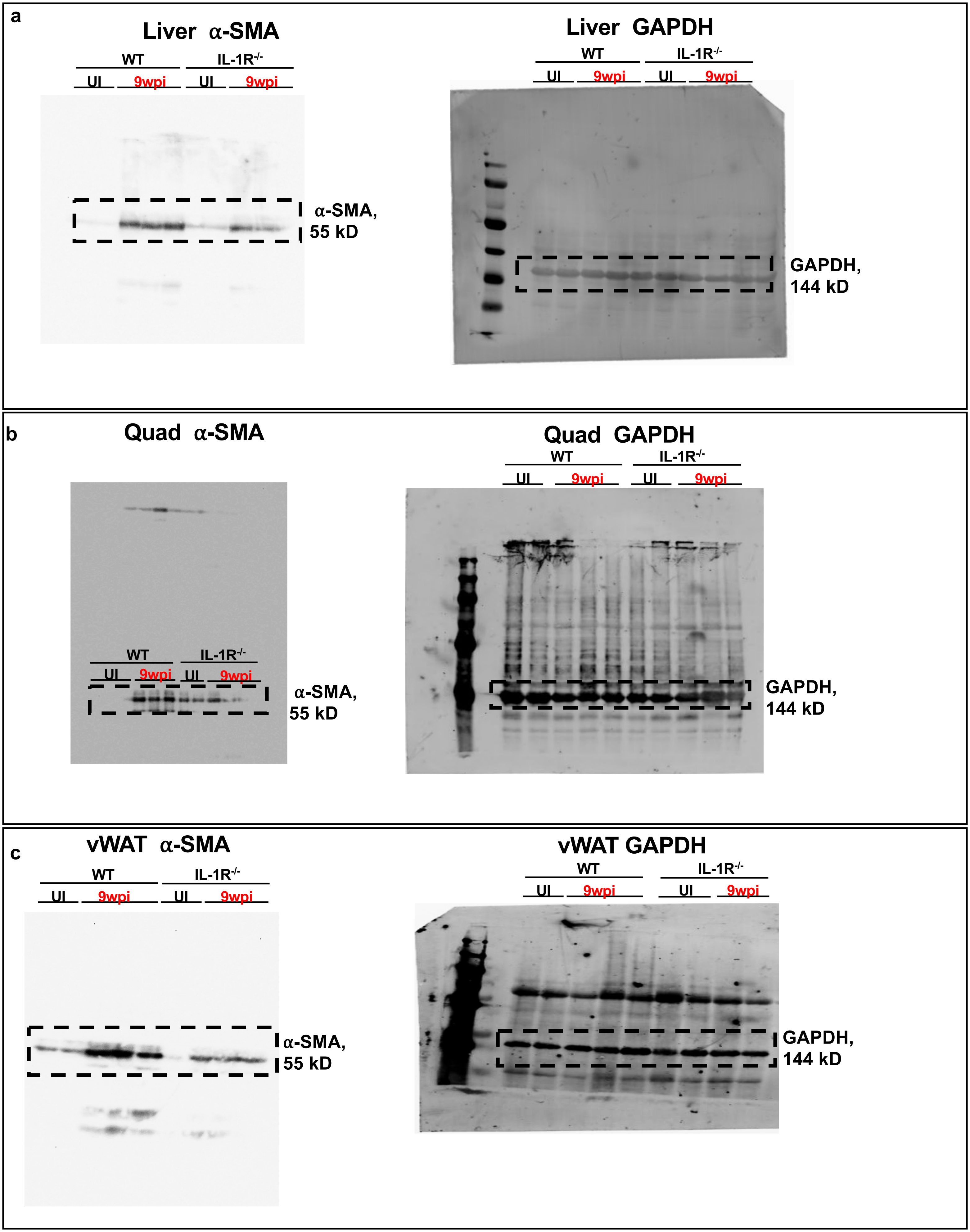

Supplementary Figure 7
